# Biological Potential and Chemical Profile of European Varieties of *Ilex*

**DOI:** 10.3390/foods11010047

**Published:** 2021-12-25

**Authors:** Natalia Pachura, Robert Kupczyński, Jordan Sycz, Agata Kuklińska, Anna Zwyrzykowska-Wodzińska, Katarzyna Wińska, Aleksandra Owczarek, Piotr Kuropka, Renata Nowaczyk, Przemysław Bąbelewski, Antoni Szumny

**Affiliations:** 1 Faculty of Biotechnology and Food Science, Wrocław University of Environmental and Life Sciences, Norwida 25, 50-375 Wroclaw, Poland; jordan.sycz@upwr.edu.pl (J.S.); katarzyna.winska@upwr.edu.pl (K.W.); antoni.szumny@upwr.edu.pl (A.S.); 2 Department of Environment, Animal Hygiene and Welfare, Wrocław University of Environmental and Life Sciences, Chełmońskiego 38C, 51-630 Wroclaw, Poland; robert.kupczynski@upwr.edu.pl (R.K.); agata.kuklinska@upwr.edu.pl (A.K.); anna.zwyrzykowska@upwr.edu.pl (A.Z.-W.); 3 Department of Pharmacognosy, Faculty of Pharmacy, Medical University of Lódź, Muszyńskiego 1, 90-151 Lodz, Poland; aleksandra.owczarek@umed.lodz.pl; 4 Department of Animal Physiology and Biostructure, Wrocław University of Environmental and Life Sciences, Norwida 31, 50-375 Wroclaw, Poland; piotr.kuropka@upwr.edu.pl (P.K.); renata.nowaczyk@upwr.edu.pl (R.N.); 5 Department of Horticulture, Wrocław University of Environmental and Life Sciences, Grunwaldzki Square 24A, 50-363 Wroclaw, Poland; przemyslaw.babelewski@upwr.edu.pl

**Keywords:** *Ilex*, *Ilex aquifolium*, terpenoids, Wistar rats, regulation of lipid metabolism

## Abstract

Plants of the genus *Ilex* are widespread throughout the world, with its best-known representative being *Ilex paraguraiensis* from South America. The European species *Ilex aquifolium* shows similarities in its terpenoid, sugar and phenolic acid profiles. Using aqueous extracts of *Ilex aquifolium* as a supplement in Wistar rats showed that, despite the lack of caffeine, it had strong hypocholesterolemic effects. In addition, a reduction in oxidative lipid degradation and a decrease in hepatic steatosis in histopathological studies were observed. The results of this study suggest that extracts from the European species *Ilex aquifolium* may have potential as an alternative treatment for hyperlipidemia.

## 1. Introduction

Plants contain a high number of secondary metabolites, some of which possess several beneficial properties for human health. These substances are referred to as secondary metabolites and perform functions that are still not fully understood [1,2]. For centuries, herbs have been used to make medicinal beverages to treat many symptoms and diseases in the human body [3]. Some herbs are well known for their biological properties that may be useful in the fight against metabolic syndrome diseases such as diabetes and dyslipidemia [4]. The chemical composition and medical uses of plants belonging to the Aquifoliaceae family (of the genus *Ilex*) have been studied in this regard. Certain parts of these plants, such as the leaves and the stems, include many bioactive substances, such as saponins, polyphenols, glycosides, alkanes, esters, ketones, aldehydes, and lipophilic compounds [5,6].

The genus *Ilex* comprises approximately 810 species growing and occurring in the temperate and subtropical climates of both hemispheres [7]. The most significant diversity of the *Ilex* species is found in South America and East Asia. Several taxa are also found in the temperate climates of Europe and North America, and a few others can be found in Australia and Africa [8]. One of the most popular species is *Ilex paraguariensis* A.St.-H, an evergreen tree or shrub that grows in temperate or tropical climates in southern Brazil, in the northeast of Argentina, and in Paraguay [6,9]. This plant has great functional importance in South America and is thus a comparative species due to its well-documented composition and biological properties [10,11] and it is used to make traditional infusions processed from leaves and twigs called “Yerba mate”, “erva mate” or “chimarrão” [12,13]. This herbal product has gained great cultural significance and is a daily beverage consumed by people in South America [14]. The per capita consumption of Yerba mate in South America is up to one liter of the brew per day [15]. Mate’s popularity is growing outside of South America as a functional food that has been shown to be beneficial for human health. The well-studied chemical composition and biological properties of the popular Yerba mate (*I. paraguariensis var. paraguariensis*) have prompted researchers to look at other species of the genus *Ilex* for an alternative pharmacognostic source [15,16,17].

The European species of the genus *Ilex* are cultivated for their high ornamental value and year-round foliage [18]. A few selected species of *Ilex* (mainly *I. aquifolium*), which have distinctive red fruits and jagged leaves, are referred to as “holly” [19]. Botanically, these plants occur as shrubs or small trees with distinctive green leaves and inconspicuous flowers. Many cultivars and hybrids have been developed for horticultural use; for example, the ”Argentea Marginata” with white leaves or “Bacciflava” with an attractive yellow fruit [20]. In Poland, the holly genus does not occur on natural stands but, due to the transitional climate, it has been cultivated as several species with evergreen leaves, such as *I. aquifolium* L. and the hybrid *Ilex × meserveae* S.Y.Hu. These are wintering species that grow best in the warmest regions of western and south-western Poland, which are characterized by relatively mild winters.

*Ilex aquifolium* grows in temperate and subtropical climates, inhabiting mainly western Europe, from Spain and France to western Norway, the Atlas Mountains in Africa, and the northern parts of Turkey and the Caucasus [21,22]. In Poland, it has the form of a small tree or a high shrub, reaching a maximum of seven meters in height and forming a dense pyramidal or elongated crown. Shrubs of this species are considered inductor plants in central Europe for climate subzone seven, as suggested by Heinze and Schreiber [23]. The second commonly planted and cultivated species is *I. × meservea*, a hybrid between *I. aquifolium* and *I. rugosa* F. Schmidt, originating from Japan and Sakhalin, Russia. Varieties of this species have been cultivated since 1960 in Canada. They are considered to be more resistant to low temperatures than *I. aquifolium*. They form dense shrubs reaching approximately two meters in height with dark green glossy leaves with a matte blue tone. There are 36 cultivated varieties of this hybrid in gardens all over the world [24].

The chemical constituents of the South American *Ilex* species are quite extensive. Although the European species have been well characterized botanically, knowledge of the biological properties of each fraction substance, as compared to the South American species, is incomplete [25]. Based on the origin of different biochemical pathways, the metabolites produced by plants can be divided into three chemical groups: terpenoids, alkaloids, and phenolic compounds [26,27]. A phytochemical analysis of Yerba mate revealed the presence of a dominant fraction of polyphenols that include caffeoyl derivatives such as caffeic acid, chlorogenic acid, 3,4-dicaffeoylquinic acid, 3,5-dicaffeoylquinic acid, and 4,5-dicaffeoylquinic acid [28,29]. Additionally, flavonoids such as quercetin, kaempferol, naringenin, and rutin have also been reported [30]. Another class of compounds identified was xanthines such as caffeine, theophylline, and theobromine [31,32,33]. Numerous triterpenoid saponins have also been identified, including those derived from ursolic or oleanolic acids, and most of them occur as glycosides [34].

Phytochemical studies performed on *I. aquifolium* revealed the presence of various secondary metabolites such as terpenoids, anthocyanins, flavonoids, and sterols [6,35]. In preliminary studies conducted by Zwyrzykowska et al. [25] and Kuropka et al. [36], the European *Ilex* species was shown to contain a high amount of polyphenolic fractions such as rutin, quinic acid, and its caffeoyl esters, glycosidic triterpenes. A similarity in chemical composition was found between the cultivar *I. meserveae* (i.e., ‘Blue Angel’) and the South American *I. paraguariensis*. In other studies conducted on plants’ antioxidant properties, quercetin from the leaves and dihydroxyphenylacetic acid and its methyl esters isolated from the seeds of *I. aquifolium* showed the ability to scavenge free radicals [35]. Recent studies conducted on aqueous extracts prepared from *I. meserveae* confirmed its use as a food preservative [37].

In folk medicine, a decoction of the leaves of the *I. aquifolium* was used to treat intermittent fevers and to relieve rheumatic pains. The plant was considered an antipyretic, an analgesic, a diuretic, and an expectorant [38]. In the study conducted by Nurgün Erdemoglu et al. [39] *I. aquifolium* leaf extracts isolated by organic solvents showed moderate antibacterial activity against *Escherichia coli*, *Staphylococcus aureus*, *Pseudomonas aeruginosa*, *Enterobacter aerogenes*, *Proteus vulgaris*, *Salmonella* Typhimurium, and *Candida albicans*. However, the fruits of the *Ilex* may be toxic to humans [40]. Numerous studies conducted using animal models have provided evidence that the plant may have potential in the treatment of the “diseases of civilization” [41]. The health-promoting effects of Yerba mate have been attributed to antioxidant, hypocholesterolemic, hypotensive, anti-diabetic, and chemopreventive properties [42,43,44].

Consumption of mate tea has been shown to improve serum lipid parameters in healthy individuals with dyslipidemia. The hypolipidemic effect is attributed to the inhibition of pancreatic lipase activity, as has been shown by studies in vivo with an obese mouse model [45]. Studies on metabolic disease syndrome, including type 2 diabetes, have shown that an infusion derived from *I. paraguariensis* may affect glycemic control and lipid profile [46]. A mate extract reduced insulin resistance in mice on an obesity-inducing high-fat diet [47]. In vivo studies showed that a mate extract exhibited anti-obesity effects. A reduction in lipid accumulation in adipocytes was observed, leading to slower fat gain [30]. The biological activity of polyphenol fractions should be noted at this point. It has been shown that the presence of polyphenolic compounds in the ingested diet reduces the risk of obesity, cardiovascular disease, and type 2 diabetes [48,49,50].

In the present study, the quantitative and qualitative composition of different varieties of *I. aquifolium* occurring in Europe were examined, and the one containing the highest amount of the terpenoid fraction was selected. This study aimed to evaluate the effects of aqueous extracts of selected *Ilex* species and the terpenoid fraction on hyperlipidemia induced in Wistar rats fed a high-fat diet, as well as on the metabolism of their action. It was hypothesized that terpenoids extracted from *Ilex* may affect the antioxidant status and histological changes in the liver. Additionally, we explored whether *I. aquifolium* could be a substitute for the well-known yerba *I. paraguariensis,* which has well-established hyperlipidemic properties.

## 2. Materials and Methods

### 2.1. Plant Material

Fresh leaves of *I. aquifolium* were obtained from Wrocław University of Life Sciences and were from their own cultivation (Vegetable and Ornamental Plant Research and Education Station, Psary, Poland, 51°11′05″ N 17°01′53″ E). Shrubs of holly *I. aquifolium* were grown in the ornamental plant collection of the Department of Horticulture on medium loam humus soil covered with pine bark mulch. The mean annual temperature was 10.1 °C. The maximum temperature was 32 °C and the minimum was −16.9 °C. The warmest month was July, with an average of 20.5 °C and the coldest month was January, with −0.6 °C. Annual precipitation averaged 468.8 mm. The highest precipitation was recorded in July—80.8 mm—and the average humidity was about 28%. After harvesting, the selected cultivars were precleaned to remove contaminants and then subjected to a freeze-drying process. The freeze-drying process was carried out for 48 h using a Lyovac GT 2 apparatus. The dried leaves were then crushed to obtain homogeneous material for the study. The dried leaves of *I. paraguariensis* (yerba mate) were purchased from a local distributor and were used as a reference.

### 2.2. Isolation and Analysis of Terpenoid Fraction

To prepare the terpenoid fraction, 200 mg of mortar-ground plant material was macerated with 10 mL of hexane. The profile of the higher terpenoids was evaluated via derivatization using the silylation reagent N,O-Bis (trimethylsilyl)trifluoroacetamide (BSTFA) on the GC-MS (Shimadzu QP 2020, Shimadzu, Kyoto, Japan). The solution was filtered and evaporated on a vacuum evaporator (Heidolph-VAP Core) under reduced pressure. Then, 500 µL of pyridine and 50 µL of BSTFA were added to the sample. The mixture was transferred to a vial and heated for 25 min at 70 °C. Prepared samples were stored at a temperature of −18 °C until GC-MS analysis.

Analysis of all fractions described in this paper was performed using a gas chromatograph coupled to a mass spectrometer (Shimadzu GC-MS QP 2020 single-quadrupole gas chromatograph mass spectrometer, Shimadzu, Kyoto, Japan). Analyses were performed in triplicate.

Separation was achieved using a Zebron ZB-5 capillary column (30 m, 0.25 mm, 0.25 µm; Phenomenex, Torrance, CA, USA). GC-MS analysis was performed according to the following parameters. Scans were performed from 40 to 1050 *m*/*z* using electron impact ionisation (EI) at 70 eV, in a mode of 10 scans s^−1^. Analyses were performed with helium as a carrier gas at a flow rate of 1.0 mL min^−1^ at 1:20 and the following program: 100 °C for 1 min, a rate of 2.0 °C min^−1^ from 100 to 190 °C; and a rate of 5 °C min^−1^ from 190 to 300 °C. The injector was maintained at 280 °C, respectively. Compounds were identified using two different analytical methods for comparison: Retention times of authentic chemical compounds (Supelco C7-C40 Saturated Alkanes Standard) and mass spectra obtained with an available library (Willey NIST 17, fitting index > 90%).

The identification of all compounds analyzed on GC-MS was based on three independent methods—(i) comparison of the calculated retention indices (RI) using the retention index calculator with the values in the NIST 20 database, (ii) comparison of the obtained spectra with the NIST 20 databases, and (iii) comparison of the retention times (RTs) with the retention times of the authentic standards. Two programs AMDIS (v. 2.73) and GCMS solution (v. 4.20) were used to process the spectra.

### 2.3. Isolation and Analysis of Phenolic Acids Fraction

The method described by Snook et al. [51] was used to extract phenolic acids. Plant material weighing 500 mg was macerated with 20 mL of 2 M NaOH and 0.1 mg of resveratrol as an internal standard. The extract was filtered and then washed twice with 10 mL ethyl acetate. The extract was then acidified with 1 M HCl to pH = 2 and washed twice with 5 mL ethyl acetate. The acetate fraction was transferred to a separate vial and washed twice with 5 mL of water. The organic phase was dried with MgSO_4_, filtered, and evaporated to dryness on a vacuum evaporator. The sample was derivatized with 500 µL of pyridine and 100 µL of BSTFA silylation reagent by maintaining the sample in a heating block at 60 °C for 20 min. Samples were stored at −18 °C until chromatographic analysis.

The separation of the phenolic acid fraction was achieved using the Zebron ZB-5 capillary column (30 m, 0.25 mm, 0.25 μm; Phenomenex, Torrance, CA, USA). GC-MS analysis was performed under the following parameters: scans from 40 to 1000 *m*/*z* with the use of EI at 70 eV, in a mode of 10 scans s^−1^. Analyses were performed with helium as a carrier gas at a flow rate of 1.02 mL min^−1^ at 1:50 and the following program: 100 °C for 1 min, a rate of 2.0 °C min^−1^ from 100 °C to 190 °C, and a rate of 5 °C min^−1^ from 190 to 300 °C for 25 min. The injector was maintained at 280 °C, respectively.

### 2.4. Isolation and Analysis of Sugar Fraction

The method described by Becker et al. [52] was used (with minor modifications) to prepare sugar samples for GC-MS analysis. Plant material weighing 200 mg was ground in a mortar and extracted with 10 mL MeOH/H_2_O 80:20 *v*/*v*, and 0.5 mg of myo-inositol was added as an internal standard. The extract was filtered and evaporated under reduced pressure. Then, 250 µL of methylhydroxylamine-hydrochloride (NH_2_OCH_3_/HCl 25 mg/mL) was added and incubated in a water bath at 75 °C for 30 min. A total of 250 µL of silylation reagent—BSTFA—and 500 µL of pyridine were added to the mixture and incubated in a water bath at 60 °C for 20 min. Samples were stored at a temperature of −18 °C until GC-MS analysis.

The separation of sugar fraction was also achieved using the Zebron ZB-5 capillary column (30 m, 0.25 mm, 0.25 μm; Phenomenex, Torrance, CA, USA). The program parameters were as follows: scans from 40 to 1050 *m*/*z* with the use of EI at 70 eV, in a mode of 10 scans s^−1^. The carrier gas during the analysis was helium, and its flow rate was set at 1.0 mL min^−1^ with a 1:20 split ratio and the following program: 150 °C for 1 min, a rate of 1.0 °C min^−1^ from 150 °C to 180 °C, and a rate of 4 °C min^−1^ from 180 °C to 300 °C for 10 min. The injector was maintained at 280 °C, respectively.

### 2.5. Isolation and Analysis of Caffeine and Theobromine

#### 2.5.1. GC-MS Analysis

For the extraction of caffeine and theobromine, the method described by Negrin et al. [8] was used with minor modifications. In brief, 100 mg of dry, powdered plant material was macerated for 3 h in 3 × 2 mL of chloroform and shaken. Then, the organic phase was evaporated, filtered through celite (50 mg), and finally dissolved in 100 µL of chloroform. The mixture was transferred to an insert vial. It was stored at −18 °C until GC-MS analysis.

Caffeine and theobromine were identified when the sample was injected onto a Zebron ZB-5 capillary column (30 m, 0.25 mm, 0.25 μm; Phenomenex, Torrance, CA, USA). Analysis was performed using SIM mode (*m*/*z* ions for caffeine 194 and 109, and for theobromine 180 and 109) and the program was as follows: 150 °C for 1 min, a rate of 10.0 °C min^−1^ from 150 °C to 250 °C for 3 min. The other parameters of the analysis were as shown below: electron impact ionization (EI) at 70 eV, split ratio 1:100, column oven at 150 °C, injector temperature at 260 °C. Analyses were performed with helium as a carrier gas at a flow rate of 1.01 mL min^−1^.

#### 2.5.2. LC-MS Analysis

In the LC-MS approach, dry, powdered plant material was extracted 3 h in 3 × 2 mL of chloroform. Then, after filtration, organic residue was dissolved in water:methanol 1:1, and subjected to LC-MS analysis. A calibration curve was also prepared for caffeine and theobromine in the range of 10–200 ng/mL.

LC-MS analysis of caffeine was performed using a high-performance liquid chromatography system (HPLC, Shimadzu Prominence-i LC-2030C) coupled to a triple quadrupole mass spectrometer (Shimadzu LCMS-8045). Separation was achieved on a reversed-phase column (Kinetex 2.6 µm C18 100A, 100 × 3 mm, Phenomenex, Aschaffenburg, Germany) at 35 °C. Two mobile phases were used during the analysis: water with 0.1% formic acid (eluent A) and methanol with 0.1% formic acid (eluent B). The flow rate was set at 0.3 mL/min and the gradient was as follows: starting with 20% solvent B for one minute, then to 90% B until 6 min, then 90% B for 2 min, then to 20% B until reaching 12 min, and finally 20% an until 15 min. The injection was 1 µL.

The electrospray ionization (ESI) was operated in positive mode and the ion spray voltage was set at 4000 V. Caffeine and theobromine were analyzed via multiple reaction monitoring (MRM) with the parameters given in Table 1. The other MS parameters were determined as follows: nebulizing gas flow 3 L/min, heating gas flows 10 L/min, interface temperature 300 °C, desolvatation temperature 526 °C, heat block temperature 400 °C, and drying gas flow 10 L/min.

### 2.6. Animal Model—Maintenance and Feeding of Animals

The animal study protocol was approved by the Local Ethics Committee (decision No. 94/2015, Wrocław, Poland) before the onset of the trial. Six-week-old Wistar male rats (n = 48) were purchased (Harlan Laboratories, Madison, WI, USA) and fed a standard chow diet for a week and a half to acclimate them to their new environment. At eight weeks of age, forty Wistar rats were randomly allocated to five groups (n = 8 animals per group): rats fed with standard feed (CON group), rats receiving 50 g/mL *I. aquifolium* extract (ILEX group), rats receiving 10 mg/kg body weight of the terpenoid fraction extracted from *I. aquifolium* (TERP group), rats receiving feed supplemented with 20 g cholesterol/kg of standard feed (CHOL group), and rats receiving 10 mg/kg body weight of terpenoid fraction supplemented with 20 g cholesterol/kg of standard feed (TERPCHOL group). The extracts were combined with water and administered ad libitum. The duration of the experiment was 8 weeks. The doses of active substances were selected according to de Resende et al. [53].

The animals were kept in a conventional animal house. The temperature during the experiment was 22 °C ± 2 °C. The room was air-conditioned (15 cycles per hour) and the relative humidity was 55%. The animals were provided with access to natural light on a 12 h day/night schedule. The rats were maintained in standardized cages 378 × 217 × 180 mm, two rats per cage. The surface area of each cage was 820 cm^2^. All cages were provided with water bottles, feeders, water, and feed. The arrangement of the cages in the test room allowed the rats visual, auditory, and olfactory contact. The rats used in the experiment were fed a complete standard feed in pellet form, the composition of which is shown in Table 2. below. Gross energy was measured using an adiabatic bomb calorimeter (KL 12 Mn, PPHU, Poland) and chemical analysis of pellets was performed according to AOAC recommendations (2005). The rats were checked every day and weighed twice a week. Food intake was monitored and the rats were weighed twice per week. After 8 weeks of feeding, the rats were euthanized to collect blood and tissue samples.

### 2.7. Blood Biochemical Analyses

Blood was collected in sterile test tubes for serum (Sarsted, Blizne Łaszczyńskiego, Poland), in tubes with anticoagulant (EDTA-K3; Sarsted, Poland), in tubes containing EDTA (VT-100 STK, 0.1 mL of EDTA, 0.47 mL/L: 21 *w*/*v*; CML, Nemours, France)m and in tubes filled with heparin sodium salt (Sarsted, Poland). The blood samples for serum or plasma were centrifuged at 3000× *g* for 10 min at room temperature (two hours from collection), and the samples were frozen (−20 °C) until the analysis.

Analysis of hematological parameters was performed using the ABC Vet analyzer (Horiba ABX Diagnostics, France). Parameters such as red blood cell (RBC), white blood cell (WBC), platelets (PLT), hemoglobin (HGB), hematocrit (HCT), mean corpuscular volume (MCV), mean corpuscular hemoglobin (MCH), mean corpuscular hemoglobin concentration (MCHC), lymphocytes (LYM), monocytes (MON), and granulocytes (GRA) were recorded.

Biochemical studies were performed using a Pentra 400 (Horiba ABX Diagnostics, France). The following were estimated parameters: nonesterified fatty acids (NEFA) via the enzymatic method, reagents Randox (Crumlin, Dublin, Ireland); triglycerides (TG) and total cholesterol by enzymatic methods, reagents HORIBA ABX (Montpellier, France); high-density lipoprotein cholesterol (HDL) and low-density lipoprotein cholesterol (LDL) by means of a colorimetric assay (Horiba ABX, Montpellier, France), the glucose oxidase method (Horiba ABX, Montpellier, France); aspartate aminotransferase (AST), lactate dehydrogenase (LDH) and thr creatine kinase (CK) activity-to-enzymes kinetic method, HORIBA ABX reagents (Horiba ABX, Montpellier, France); creatinine through the enzymatic method (Horiba ABX, Montpellier, France); total protein (TP) and albumin (Alb) via a colorimetric method (Horiba ABX, Montpellier, France).

The following parameters of antioxidant status were also determined. Total antioxidant capacity (TAS) in serum, using the colorimetric method based on ABTS (2,2′-azine-di-[3-ethylbenzothiazoline sulfate]) with peroxidase—glutathione peroxidase (GPx) in whole blood via the enzymatic method, and—superoxide dismutase (SOD) in erythrocytes by the spectrophotometric method based on a reaction with 2-(4-iodophenyl-3-(4-nitrophenol)-5-phenyltetrazoline chloride (INT). The concentration of malondialdehyde (MDA) in blood serum using the bovine malondialdehyde (MDA) ELISA kit from MyBioSource (San Diego, CA, USA). Immunological parameters were determined in serum. Serum IgG immunoglobulin was assayed using the Bethyl Bovine IgG ELISA Kit. Serum IgM immunoglobulin was determined using a Bovine IgM ELISA Kit, Bethyl (Montgomery, AL, USA). Serum IL-6 and IL-10 interleukin were assayed using an ELISA Kit immunoassay from MyBio Source (San Diego, CA, USA). These measurements were conducted using a Synergy fluorescence, luminescence, and absorbance reader from BioTek Instruments (Winooski, VT, USA).

### 2.8. Isolation and Analysis of Fatty Acid Methyl Esters (FAME)

The method described by Folch et al. [54] was used to extract lipids from liver, blood serum, and urine samples.

#### 2.8.1. Wistar Rat Liver Samples

After harvesting the rat livers, they were subjected to a freezing process. Afterwards, to prepare the material for further steps, they were subjected to a 24 h lyophilization process on a Lyovac GT 2 apparatus. A total of 100 mg of lyophilized tissue was macerated using 5 mL of a mixture of chloroform:methanol (2:1) and heptadecanoic acid as an internal standard. The chloroform extract was filtered and evaporated on a vacuum evaporator. The non-polar fraction was then treated with 2 mL of 0.5 M KOH/MeOH solution for 5 min at 65 °C, and methylation was initiated for 5 min at 65 °C by adding 1.5 mL of 14% (*v*/*v*) BF_3_/MeOH (Sigma-Aldrich, St. Louis, MO, USA). Water and 10 mL of hexane (UQF, Wrocław, Poland) were added to the solution and fatty acid methyl esters were extracted. The mixture was washed with 10 mL of 10% sodium bicarbonate (UQF, Wrocław, Poland) and dried over anhydrous sodium sulfate. The organic phase was evaporated under reduced pressure, the material was dissolved in 200 µL hexane, transferred to an insert vial, and subjected to chromatographic analysis.

For samples prepared from rat livers, separation was achieved using a Zebron ZB-FAME capillary column (60 m, 0.20 mm, 0.20 µm; Phenomenex, Torrance, CA, USA). GC-MS analysis was conducted using the following parameters: scanning was performed from 40 to 400 *m*/*z* in electron beam ionization (EI) at 70 eV, in a mode of 3 scans s^−1^. Analyses were performed using helium as a carrier gas at a flow rate of 1.8 mL min^−1^ at 1:10 and the following program: 80 °C for 2 min; rate of 3.0 °C min^−1^ from 80 °C to 180 °C; rate of 8 °C min^−1^ from 180 °C to 240 °C. The injector was maintained at 280 °C, respectively.

#### 2.8.2. Wistar Rats Blood Plasma and Urine

The preparation of FAME samples from plasma and urine was analogous to liver samples with minor modifications. Plasma in a volume of 150 µL was transferred to a 2 mL Eppendorf tube and macerated using a solvent mixture of methanol:chloroform (500 µL:250 µL) and 10 µg of standard (heptadecanoic acid) was added. Samples were centrifuged and the organic layer was collected, transferred to a vial and evaporated. The hydrolysis process was then initiated by adding 300 µL of 0.5 M KOH/MeOH for 5 min at 65 °C. Subsequently, 100 µL of 14% (*v*/*v*) BF_3_/MeOH (Sigma-Aldrich, St. Louis, MO, USA) was added and maintained at 65 °C for 3 min to obtain the FAME, then 50 µL of water and 400 µL of cyclohexane were added to the mixture. The organic fraction was filtered and placed in a 200 µL insert and vial. The samples were placed in a freezer at −18 °C until GC-MS analysis.

FAME analysis of blood serum and urine was performed under analogous parameters. A modification of the method was the use of SIM mode, where the presence of the following *m*/*z* ions was monitored: 55.0, 74.0, 79.0, 81.0, 87.0, 143.0.

### 2.9. Histopathological Examination of Tissues

After collection, the material was fixed in a 4% buffered formalin solution and then dehydrated in an alcohol series and embedded in paraffin. Sections 5 µm thick were routinely stained with hematoxylin and eosin. Morphological analysis concerned the presence of the steatosis, hepatocytes damage, edema, and leukocyte infiltration, as well as the status of bile synthesis by hepatocytes. The examination was performed using a Nikon Eclipse 80i light microscope.

### 2.10. Statistical Analyses

Statistical analyses were performed on Statistica version 13.3 (StatSoft, Kraków, Poland). In vivo samples were analyzed in duplicates; the values for each group represent the mean of eight animals (n = 8). All the results were expressed as means and standard deviation, and all variables were tested for normality using the Shapiro–Wilk test. The data obtained were subjected to analysis of variance (ANOVA) using a linear model procedure. The significance of differences between the test results was determined by means of the Tukey test. Effects were considered significant at a probability of *p* < 0.05. The remainder of the analyses were subjected to the analysis of variance using Duncan’s test (*p* < 0.05). Principal component analysis (PCA) was carried out based on the correlation matrix of the quantitative data obtained for the investigated samples.

## 3. Results and Discussion

The results presented in this paper indicate the similarity of Argentine *I. paraguariensis* with European varieties of *I. aquifolium*, from chemical constituents to potential biological properties. Our work is one of the first to explore the actual terpenoid, sugar, and phenolic acid contents of *I. paraguariensis* and European varieties of *I. aquifolium*.

### 3.1. Terpenoid Fraction

Analysis of the terpenoid fraction isolated from the European species *I. aquifolium* revealed the presence of 14 compounds (a table with all compounds is available in the Supplementary Data), of which the 10 main ones are presented in Table 3. These include α-amyrin (2.43–4.89 mg/g), lupeol (2.77–1.20 mg/g), betulinic alcohol (2.19–0.99 mg/g), β-amyrin (1.79–0.93 mg/g), ursolic aldehyde (1.05–0.32 mg/g), and ursolic acid (0.84–0.36 mg/g). For comparison, the table also shows the results of triterpenes identified in *I. paraguariensis*, showing the similarity of the Argentine *Ilex* variety with the European one. In contrast, lower amounts of uvaol, betulinic acid, and oleanic acid, as well as higher contents of lupeol and ursolic aldehyde, were observed in *I. aquifolium*, as compared to *I. paraguariensis*.

The compounds with biological activity from the triterpene group have also been identified in other *Ilex* cultivars. In the leaves of *I. cornuta* lupeol, betulonic acid, uvaol, ursolic acid, and α-amyrin were identified. In *I. centrochinensis*, the presence of lupeol and oleanic acid was revealed. Equally, lupeol, betulinic alcohol, ursolic acid and uvaol were also identified in the Chinese variety *I. latifolia* [48]. In addition, lupeol and betulin isolated from the leaves of *I. macropoda* have been shown to inhibit the activity of a human acyltransferase enzyme involved in cholesterol metabolism [55]. This evidence suggests the similarity of *Ilex* cultivars among plants found in different parts of the world, which may also indicate similar biological properties. With regard to the biological properties of the terpenoid fraction, analogous compounds have already been identified in earlier studies that focused on medicinal plants. Investigations carried out on dandelion leaves have revealed comparable contents of components such as β-sitosterol, α-amyrin, and β-amyrin [49], where in the leaves of *Ilex* species, the amounts of these same components decreased as follows: α-amyrin, β-amyrin, and β-sitosterol. Siddiqui et al. [50] identified the presence of bioactive terpenoids in the hibiscus plant; however, the authors focused on validating a method to determine oleanolic acid and β-amyrin in different fractions. In sage leaves, the representative triterpenoids are pentacyclic triterpene acids such as oleanic acid, ursolic acid, and betulinic acid [56]. Niżinski et al. [57] studied marigold leaves and identified the presence of α-amyrin, β-amyrin, and lupeol, whereas no oleanic acid was identified. Amyrin-rich raw materials include several herbs, i.e., *Byrsonima crassifolia* (0.9 mg/g), Mexican copal (0.5 mg/g), and *Amphipterygium adstringens* (2.4 mg/g) [49]. Amyrine fractions, as well as the pure compounds, possess proven antihyperglycemic and hypolipidemic activity. A dose of 10 mg/kg b.w. α-amyrines and β-amyrines, isolated from *Protium heptaphyllum* significantly decreased TC and TG in serum [58,59].

### 3.2. Phenolic Acid Fractions

The phenolic acid profile of European *I. aquifolium* showed 31 phenolic acids, of which eight of the major acids are summarized in Table 4 (a table with all compounds is available in the Supplementary Data). In Table 4, caffeic acid (2.88–1.64 mg/g) is the main representative, followed by malic acid (0.55–0.07 mg/g), quinic acid (0.22–0.01 mg/g), and ferulic acid (0.20–0.06 mg/g). For comparison, the phenolic acid profile of *I. paraguariensis* was also studied, thus proving the similarity in the phenolic acid profile of the Argentine variety with that of the European variety. Lower amounts of 3-O-Coumaroyl-D-quinic acid, 4-O-Caffeylquinic acid, and chlorogenic acid were observed in the *I. aquifolium* cultivars, as compared to *I. paraguariensis*, as well as higher amounts of quinic acid and malic acid.

Phenolic acids, known for their antioxidant properties, are also a widely recognized group of active substances. Bojić et al. [30] identified the following bioactive compounds in *I. paraguariensis*, such as chlorogenic acid (2.1 mg/g) and caffeic acid (1.5 mg/g). In the same plant, Vieira et al. [60] also confirmed the presence of 4,5-di-O-caffeylquinic acid, chlorogenic acid, gallic acid, p-coumaric acid, caffeic acid, and ferulic acid. In other *Ilex* species, researchers have identified the presence of phenolic acids. Wang et al. [61] demonstrated the presence of hydroquinone, protocatechualdehyde, dihydroxyacetophenone, and 4,5-di-O-caffeylquinic acid in *I. pubescens*. In *I. asprella*, He et al. [62] identified caffeic acid and coniferyl aldehyde. Phenolic acids possessing antioxidant activity have been characterized in many plants used in medicine and as ingredients to improve food quality. Wojdyło et al. [63] reported that in *Echinacea purpurea* leaves, caffeic acid (6.2 mg/g), neochlorogenic acid (1.15 mg/g), p-coumaric acid (0.20 mg/g), and ferulic acid (0.19 mg/g) were identified, and in *Tanacetum vulgare* leaves, the presence of caffeic acid (8.94 mg/g), neochlorogenic acid (3.35 mg/g), and ferulic acid (0.09 mg/g) were confirmed. In other studies, Arceusz and Wesolowski [64] showed the content of phenolic acids existing in *Melissa officinalis* from different manufacturers, in which rosemarinic acid (48.6 mg/g) was found in the highest amount, followed by ferulic acid (1.59 mg/g), caffeic acid (0.71 mg/g), syringic acid (0.53 mg/g), chlorogenic acid (0.33 mg/g), and gallic acid (0.67 mg/g).

### 3.3. Sugar Fraction

Analyzing the sugar profile of various *Ilex* cultivars, 11 compounds were identified and these are presented in Table 5. The main component in the Argentine species *I. paraguariensis* and the European *I. aquifoloium* turned out to be sucrose. It was also observed that both isomers of maltose and cellobiose were absent in *I. paraguariensis* but were present in the European species. With regard to the total composition of the individual sugars, it was observed that the two European varieties, namely, *I. aquifolium* (8.57 mg/g) and *I. aquifolium* Alaska (8.78 mg/g), show a very similar sugar fraction composition, with the variety *I. aquifolium* Ferox Argentea showing a much lower sugar content in the leaves (4.82 mg/g).

When researching the sugar fraction, Maria-Ferreira et al. [65] showed the involvement of monosaccharides that were derived from the hydrolysis of soluble polysaccharides from the leaves of *I. paraguariensis*. The following sugars were identified during GC-MS analysis: glucose, galactose, arabinose, rhamnose, and mannose. In addition, Dartora et al. [66] identified fructose, glucose, and sucrose in *I. paraguariensis* leaves based on age and growth conditions.

### 3.4. Principal Component Analysis

To summarize and visualize the quantitative differences between the samples, PCA analysis was performed. This method allows for finding the correlated variables in the dataspace (if such variables are indeed present) and combining them into a smaller number of new variables (principal components, PC), which makes it easier for graphical presentation. The analysis was carried out using the quantitative results presented in Table 2, Table 3 and Table 4, which were found to be easily compressible; the first two PCs explained almost 90% of the variance present in the data.

**Figure 1:**
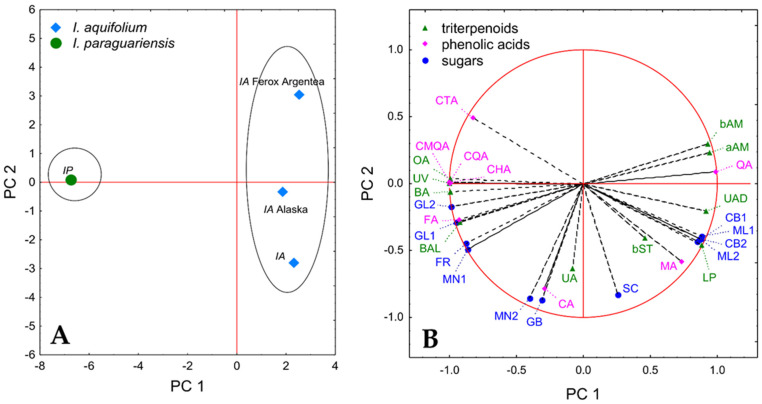
Results of the PCA analysis. (**A**) PC 1 vs. PC 2 score plots for the investigated samples. (**B**) PC 1 vs. PC 2 loading plots for the investigated variables. IA, *Ilex aquifolium*; IP, *Ilex paragurariensis*; aAM, α-amyrin; bAM, β-amyrin; bST, β-sitosterol; BA, betulinic acid; BAL, betulinic aldehyde; LP, lupeol; OA, oleanolic acid; UA, ursolic acid; UAD, ursolic aldehyde; UV, uveol; CA, caffeic acid; CHA, chlorogenic adic; CMQA, 3-O-coumaroyl-D-quinic acid; CQA, 4-O-caffeoylquinic acid; CTA, citric acid; FA, ferulic acid; MA, malic acid; QA, quinic acid; CB1, cellobiose isomer 1; CB2, cellobiose isomer 2; FR, fructose; GB, gentiobiose; GL1, glucose isomer 1; GL2, glucose isomer 2; ML 1, maltose isomer 1; ML 2, maltose isomer 2; MN 1, mannose isomer 1; MN 2, mannose isomer 2; SC, sucrose.

The first PC explained 69.90% of the variance and allowed discrimination between the *Ilex* species. The sample of *I. paraguariensis* had a negative PC 1 score, whereas all three samples of *I. aquifolium* were located on the positive side of PC 1 (Figure 1A). The loading plot for the variables (Figure 1B) indicated the constituents, of which the content is responsible for the discrimination. The results were in agreement with the trends noted for particular groups of chemicals. Among the triterpenoids, the higher contents of β-amyrin, α-amyrin, ursolic aldehyde, and lupeol were to the largest extent connected with positive PC 1 scores of the *I. aquifolium* samples. On the other hand, oleanolic acid, uvaol, betulinic acid, and betulinic alcohol had strong negative loading on PC 1; thus, higher levels of those constituents were partly responsible for the low PC 1 score of *I. paraguariensis*. The PC 1 score was also positively impacted by a higher content of quinic acid but at the same time lower by the content of its derivatives, i.e., chlorogenic acid, 4-caffeoylquinic acid, and coumaroyl quinic acid. This profile was indeed characteristic of all samples of *I. aquifolium*. Moreover, higher levels of citric acid and ferulic acid loaded negatively on PC 1. Among sugars, cellobiose and maltose isomers that were present only in *I. aquifolium* samples had high positive loading on PC1, whereas glucose isomers, fructose, and mannose isomer 1 influenced that component negatively.

PC 2 explained 19.87% of the variance. The distribution along the PC 2 axis was visible among the samples of different varieties of *I. aquifolium* (Figure 1A) and was connected mainly with differences in the content of ursolic acid, caffeic acid, sucrose, gentiobiose, and mannose isomer 2 (Figure 1B). The high content of those constituents had a negative loading on PC 2; thus, *I. aquifolium* Ferox Argentea, with a high PC 2 score, is characterized by the lowest levels of most of those chemicals.

Overall, the results of the analysis indicated some interesting trends that might be important from a chemotaxonomic point of view; however, further studies on a larger number of samples are required to investigate that topic more thoroughly.

### 3.5. Caffeine and Theobromine

In the present study, the content of caffeine and theobromine was also analyzed by means of GC-MS and LC-MS in Argentine and European varieties of *Ilex*. The presence of caffeine and theobromine was confirmed only in the case of *I. paraguariensis*, since in the European varieties of *I. aquifolium* the concentration of caffeine, as well as theobromine, was below the detection levels of the equipment used (Table 6). GC-MS analysis showed that caffeine and theobromine concentrations in *I. paraguariensis* leaves were 6.21 mg/g and 1.01 mg/g, respectively, whereas LC-MS analysis showed similar results, with caffeine at 7.77 mg/g and theobromine at 1.38 mg/g.

An important substance from the purine alkaloid family is caffeine, which stimulates the nervous system and supports the body in the process of lipolysis. In earlier work, researchers investigated the presence of caffeine only in some species of *Ilex*. The caffeine concentration in *I. aquifolium* was not investigated in previous reports. Claudia Anesini et al. [67] reported that the caffeine concentration in commercial mate tea is 13.5 mg/g. Furthermore, Kaltbach et al. [68] analyzed 19 different matte teas for caffeine concentrations and obtained results ranging from 5.63–17.57 mg/g. Three species of *Ilex* were subjected to tests for caffeine by Negrin et al. [8]. They obtained results for *Ilex paraguariensis* (4.15–11.86 mg/g), Amazonian *Ilex guayusa* (19.11–26.94 mg/g), and *Ilex vomitoria* (0.004–8.44 mg/g). Despite the lack of caffeine content in the leaves of *Ilex aquifolium*, the biological effects of the derived extracts were confirmed, which differs significantly from the findings of Zapata et al., who reported that only the caffeine content of *I. paraguariensis* leaves inhibits lipogenesis and fat accumulation and was responsible for the expression of the fatty acid synthase (FASN) and the microsomal TG transfer protein. When Zapata et al. administered the decaffeinated extract of *I. paraguariensis* in a Sprague Dawley rat model, no biological effect was observed [69].

### 3.6. Hematological and Biochemical Parameters of Blood

Prior to the study, the following groups of rats were selected to monitor blood biochemical parameters: a control group fed a standard diet without any additions (CON), a group fed a high-cholesterol diet (CHOL), a group fed a standard diet with the addition of an aqueous extract of *I. aquifolium* (ILEX), a group fed a standard diet with the addition of terpenoid fractions (TERP), and a group fed a high-cholesterol diet with the addition of terpenoid fractions (TERPCHOL).

The mean body weight gain of rats fed the cholesterol-supplemented diet was significantly higher (*p* < 0.05) than that of the rats fed a standard diet and was 86.25 ± 6.28 g for the CON group and 97.64 ± 8.62 g for the CHOL group. It was lower for the ILEX group at 77.05 ± 4.08 g, the TERP group at 79.63 ± 5.40 g, and the TERPCHOL group at 92.12 ± 4.58 g. A study by Lima et al. [53] showed that the use of Yerba mate prevented the development of hyperphagia, obesity, visceral obesity, and central leptin resistance in obese rats with early weaning. Caffeine of natural (i.e., mate and coffee) and synthetic origin promoted a reduction in fat accumulation in animals fed a high-fat, sucrose-rich diet [69]. Considering the results of our own study, the aqueous extract of *I. aquifolium* and the terpenoids extracted from it could be considered agents that decrease body weight gain.

**Figure 2:**
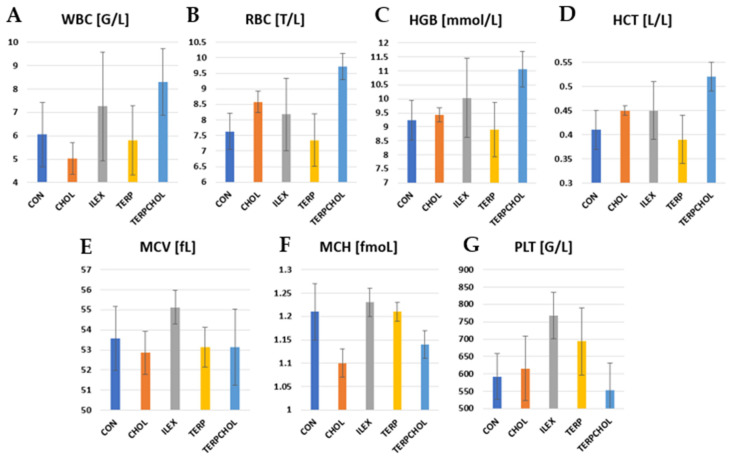
Level of (**A**) WBC, (**B**) RBC, (**C**) RBC, (**D**) HCT, (**E**) MCV, (**F**) MCH, and (**G**) PLT in CON, CHOL, ILEX, TERP, and TERPCHOL groups.

Hematological examinations are a basic indicator of metabolism and hematopoiesis. The hematological values found in the control group were in the range of values reported by other authors [70]. The administration of terpenoids in the TERPCHOL group resulted in a significant increase (*p* < 0.05) in WBC values, as compared to the control group. A similar increase (*p* < 0.05) was found after treatment with *I. aquifolium* (Table 7 and Figure 2). In contrast, there was a decrease in WBCs in the CHOL group. The supplementation, both in the ILEX and the TERPCHOL groups, caused a significant increase (*p* < 0.05) in RBCs; however, such changes did not occur after the application of terpenoids. In addition, the hemoglobin concentration was the highest in the ILEX and TERPCHOL groups. The HCT value was highest (*p* < 0.05) in the TERPCHOL group. *I. aquifolium* supplementation increased PLT, but these changes were not statistically confirmed.

**Figure 3:**
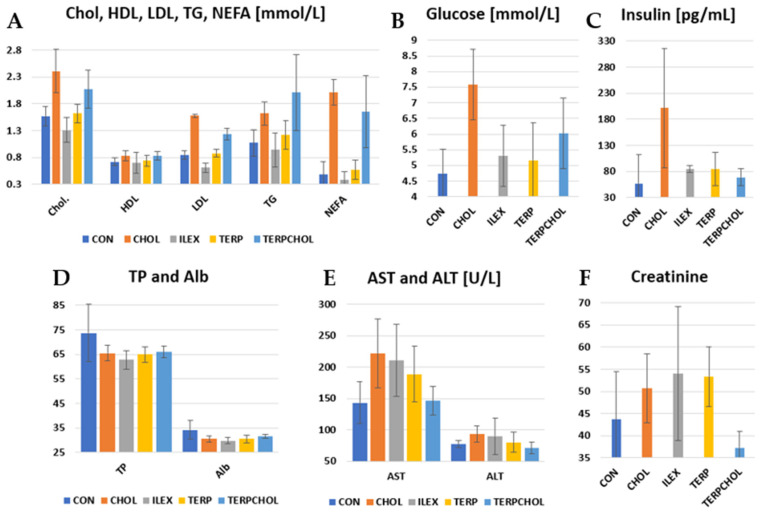
Levels of (**A**) Chol., HDL, LDL, TG and NEFA; (**B**) glucose; (**C**) insulin; (**D**) TP and Alb.; (**E**) AST and ALT and (**F**) creatinine in the CON, CHOL, ILEX, TERP, and TERPCHOL groups.

The hypercholesterolemic diet resulted in an increase in cholesterol, LDL fraction, TG, glucose, and insulin in the CHOL and TERPCHOL groups, as compared to the other groups (Table 8 and Figure 3). Changes in lipid parameters after treatment with terpenoids extracted from *I. aquifolium* were small, and the levels found were similar to those that were found in the control group. Supplementation with *I. aquifolium* resulted in a reduction in cholesterol and LDL fraction in the ILEX group, as compared to the CHOL and CON groups. The use of terpenoids reduced the serum LDL fraction, whereas a marked (*p* < 0.05) increase in TG and enhanced lipolysis was observed in the TERPCHOL group. From the perspective of the functional properties of the *Ilex* species, caffeoylquinone ester and triterpenoid saponin fractions may play a role in lowering low-density lipoprotein (LDL) levels in the blood [56,57]. An interesting relationship was observed in a study conducted by Zapata et al. [69] regarding the efficacy of phenolic compounds, specifically caffeine and caffeoylquinic acids contained in Yerba mate. The experiment involved a comparison between the isolated caffeine from mate, the whole extract containing a fraction of phenolic compounds, and the same extract devoid of caffeine. The results showed reduced in vitro lipid accumulation in rat tissues. This effect was assigned to the modulation of the FASN fatty acid synthase in 3T3-L1 adipocytes, leading to weight loss and liver fat accumulation in rats fed a high-fat, high-sucrose diet (HFSD). Preliminary in silico studies (molecular docking tests) of these substances were also performed in the context of finding potential ligands that could interact with enzymes involved in lipogenesis, as well as in lipid metabolism [71]. The administered extracts or active compounds caused an increase in blood insulin levels. There was marked hyperinsulinemia in the CHOL group, as compared to the control group (201.33 pg/mL vs. 56.67 pg/mL, respectively). *I. aquifolium* terpenoids caused a smaller increase in serum insulin concentration in the ILEX group, as compared to the CON group. The use of terpenoids with a hypercholesterolemic diet reduced the increase in blood insulin concentrations in the TERPCHOL group, indicating the effective prevention of hyperinsulinemia even when the dietary cholesterol content was high.

Feeding cholesterol to the rats resulted in an increase (*p* < 0.05) in AST and ALT activity, as compared to the control group. In addition, a similar increase occurred after supplementation with *I. aquifolium* and terpenoids in the ILEX and TERP groups. However, AST activity decreased (*p* < 0.05) with the hypercholesterolemic diet. The TERPCHOL group also had the lowest creatinine concentrations. The concentrations of TP and Alb were lower in the experimental groups, as compared to the control group.

The daily dose of cholesterol used in our study resulted in moderate hypercholesterolemia. This is supported by [72], where similar levels of total blood cholesterol were reported with a hypercholesterolemic diet. There are a number of methods to lower cholesterol, one of which is to adjust the proportion of protein and fiber in the diet, as well as the use of probiotics or phytobiotics [73,74,75]. Hypercholesterolemic rats are characterized by low LDLR gene expression and high SREBP-1c protein expression in the liver [76]. An increase in plasma FFA is one of the strongest signals stimulating the liver to synthesize triglycerides and other lipids [77]. These processes are associated with a decrease in fat mass. In our study, these relationships were also present in the CHOL group pups. The use of terpenoids with the hypercholesterolemic diet reduced the intensity of the lipolysis. In a previous study, a high-fat diet and supplementation with Yerba mate extracts from *I. paraguariensis* St. Hil. in rats prevented endothelial dysfunction by increasing nitric oxide production and regulating the expression of genes responsible for lipid metabolism [78]. Other studies have found no reduction in serum lipids with the consumption of Yerba mate (*I. paraguariensis*), polyphenols, or saponins from mate [79]. Zapata et al. [69] indicated that caffeine had been responsible for the effects of Yerba mate tea on adipogenesis and lipogenesis. However, no differences were found between total cholesterol and triglyceride levels among the groups used in that study. However, the literature indicates that differences in the preparations, doses, or timing of *Ilex* spp. aqueous extracts and their administration may be responsible for various effects on the metabolic response [78,79]. In our study, the use of the *I. aquifolium* extract lowered blood cholesterol levels, reduced lipolysis, and had a beneficial effect on glucose and insulin levels. Moreover, in other studies in which mate tea was used, it prevented an increase in insulin and decreased HDL cholesterol [80].

In recent years, a number of plants and plant extracts have been used in the prevention or treatment of diseases of civilization. Plant extracts contain large amounts of antioxidants that can play an important role in adsorbing and neutralizing free radicals, quenching oxygen, or decomposing peroxides [81,82]. Plants rich in flavonoids (e.g., ginger, turmeric, anise, coriander, and green tea), as well as anthocyanins, have antioxidant activity [83]. The composition of the active compounds depends on the type of plant. In an extract from *Rubia tinctorum* L., the antioxidant activity may be due to vanillin, rosmarinic acid, quercetin, catechin, syringic acid, or cinnamic acid [82]. In studies conducted in animal models involving obesity-inducing diets and associated insulin resistance, supplementation with Yerba extracts was shown to alleviate hyperglycemia and improve insulin sensitivity and plasma lipids [84].

**Figure 4:**
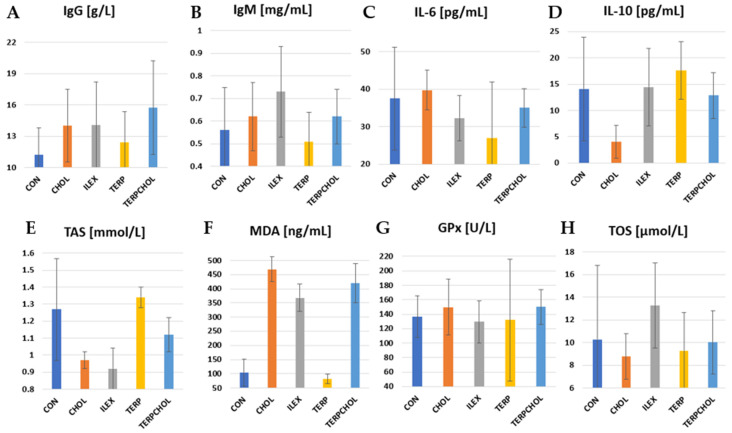
Levels of (**A**) IgG; (**B**) IgM; (**C**) IL-6; (**D**) IL-10; (**E**) TAS; (**F**) MDA; (**G**) GPx, and (**H**) TOS in CON, CHOL, ILEX, TERP, and TERPCHOL groups.

The mean values of immunological parameters and selected parameters of antioxidant status are summarized in Table 9 and in Figure 4. IL-10 is recognized as an anti-inflammatory cytokine. It inhibits the production of pro-inflammatory cytokines such as IL-2, IL-3, and TNF-α. There was a significant (*p* < 0.05) reduction in IL-10 levels in the CHOL-treated group compared to the other groups. Terpenoids caused an increase in the value of this parameter, whereas in the ILEX group, the concentration of IL-10 was similar to that found in the CON group. The lowest TOS value was recorded in the CHOL group, whereas the highest value was recorded in the ILEX group. The application of terpenoids with a hypercholesterolemic diet resulted in an increase in TOS values in the TERPCHOL group, as compared to the CHOL group. *I. aquifolium* had no effect on the blood concentrations of GPx, whereas the administration of cholesterol only or cholesterol together with the terpenoid fraction increased GPx concentrations in the CHOL and TERPCHOL groups. However, these changes were not statistically confirmed. The hypercholesterolemic diet had no significant effect on the blood concentration of IL-6. The greatest reduction in IL-6 was found after treatment with the terpenoid fraction, as well as a similar but slightly weaker effect from supplementation with *I. aquifolium*. The increase (*p* < 0.05) in MDA levels in the CHOL group indicated that oxidative stress occurred sufficiently to cause free radical-mediated lipid peroxidation in the cell membrane. A marked reduction (*p* < 0.05) in MDA was caused by terpenoid supplementation; however, the terpenoid fraction with the hypercholesterolemic diet did not reduce the increase in the MDA concentration. The use of *I. aquifolium* had no beneficial effect on MDA concentrations. The greatest increase in IgG was found in the TERPCHOL group. The IgM concentration was statistically equal (*p* < 0.05) between the ILEX and TERP groups.

Hypercholesterolemia is a risk factor of cardiovascular disease (CVD), type 2 diabetes mellitus, and metabolic syndrome [85]. Furthermore, some oxidative stress markers such as MDA increase in degenerative diseases such as diabetes mellitus [86]. In our study, TOS, GPx, and MDA levels were reduced after supplementation with terpenoids alone. Surprisingly, *Ilex* extract increased the level of serum MDA. This could be caused by other compounds, which have not been investigated in our experiment. An increasing liver MDA concentration during the administration of *I. paraguariensis* water extract was also observed by Bravo [87].

However, long-term hypercholesterolemia cannot be alleviated by the effects of terpenoids. The efficacy of these compounds has only been demonstrated in animals without metabolic disorders. In studies using hypercholesterolemic rats consuming a high-cholesterol diet, mate consumption had no effect on HDL-cholesterol or protein carbonyls, yet it showed a marked hypolipidemic action by decreasing TG, total and LDL-cholesterol, and serum MDA levels [87]. Furthermore, *I. paraguariensis* has been shown to be capable of minimizing oxidative stress during perimenopause by modulating antioxidant defense [75]. Bassalat et al. [88] showed that *T. leucocladum* manifested antihyperglycemic and antihyperlipidemic effects and also increased the antioxidative defense system and reduced the lipid peroxidation process in experimental diabetic rats. Recent research indicates that mate from *I. paraguariensis* has a direct action on ROS accumulation and activates various molecules involved in antioxidant responses such as p-CREB, NRF2, SIRT1, and SOD2 [89]. The current study showed that high concentrations of polyphenols from mate may be useful for the prevention of retinal damage in AMD and other retinal degenerations in which oxidative stress has been a pathogenic mechanism.

### 3.7. Fatty Acids

#### 3.7.1. Liver

When we profiled the fatty acid esters found in the rat livers, 48 compounds were identified, of which the main six are shown in Table 10 (a table with all the compounds is available in the Supplementary Materials). In the rat livers, the compound that was most abundant out of all the experimental groups, including the control group, was hexadecanoic acid (12.39–20.21 mg/g), followed by 9-*trans*-octadecenoic acid (8.25–14.91 mg/g) and octadecanoic acid (4.82–9.68 mg/g). With regard to the relationship between the selected groups in the experiment, an increase in the total triglyceride content of about 24% was observed in the TERPCHOL group, as compared to the CHOL group; similarly, the TERP group showed an increase of 35%, as compared to the CON group. Increased triglyceride biosynthesis and accumulation in the liver was evident in both groups. However, a different relationship was observed in the group of rats supplemented with *I. aquifolium* extracts, as they showed a lower level of triglycerides (11%) in the liver. This indicated a reduced biosynthesis of triglycerides and consequently a lower concentration in the liver. The livers from the TERP and TERPCHOL groups had higher amounts of unsaturated acids, which have been shown to be effective in combating hyperlipidemia, improving blood circulation, and inhibiting the formation of atherosclerotic plaques.

As one of the most important organs, the liver is responsible for the synthesis of cholesterol and triglycerides. In a study dedicated to the fatty acid analysis of rat livers, Wang et al. [90] also found in total fatty acids the highest content of hexadecanoic acid (13.1%), as well as arachidonic acid (14.8%) and octadecanoic acid (10.6%). Similar results were obtained by Gudbrandsen et al. [91], who also found the highest amount of hexadecanoic acid (23.3%), linoleic acid (17.4%), arachidonic acid (17.2%), and octadecanoic acid (15.2%). In a rat study by Lin et al. [92], in which they investigated the effect of DHA in the diet, they found that hexadecanoic acid was the highest (21.25%), followed by oleic acid (18.07%) and arachidonic acid (15.28%). Our research was the first to use extracts from European varieties of *I. aquifolium* and its isolated terpenoid fractions to demonstrate the effects of these bioactive substances on the lipid profiles of rat livers. In a previous study using aqueous extracts of *I. paraguariensis*, Arcari et al. [47] showed that its bioactive compounds have an effect on the mechanisms that stimulate fat oxidation, as well as inhibiting inflammation and lipogenesis in the liver. In another study, Arcari et al. [93] demonstrated the synergistic effect of compounds found in Yerba mate extract that inhibit weight gain and visceral fat accumulation. Barroso et al. [80] also confirmed that extracts from *I. paraguariensis* may reduce hepatic steatosis due to antidiabetic, anti-inflammatory, and antioxidant properties that prevent fat accumulation in the liver.

#### 3.7.2. Blood Serum

Our analysis of the serum fatty acid profile of Wistar rats revealed 24 compounds, of which four major compounds are shown in Table 11 (a table containing all the compounds is available in the Supplementary Materials). Hexadecanoic acid (7.60–27.43 mg/dL) and octadecanoic acid (8.81–26.57 mg/dL) appeared to be the main compounds, followed by 9-*cis*-octadecanoic acid (4.55–17.93 mg/dL) and linolenic acid (2.86–13.20 mg/dL). The obtained results showed a lower number of fatty acids in the ILEX group, which is indicative of their lower secretion into the bloodstream. In relation to the CON and TERP groups, no significant changes were observed in the differential secretion of fatty acids into the blood. However, in the TERPCHOL group, a higher number of fatty acids (20%) was recorded in the rat serum, as compared to the CHOL group.

Triglycerides, as fat molecules, are especially important for the supply and storage of energy required for the proper functioning of the body. In previous studies of rat serum, Wu et al. [94] reported the presence of hexadecanoic acid (109.7 mg/dL), 9-*cis*-octadecanoic acid (56.6 mg/dL), and linolenic acid (154.8 mg/dL). Mohammadi [95] studied the effect of the *Zataria multiflora* extract on rats and found similar results for hexadecanoic acid (284 mg/dL), octadecanoic acid (76.6 mg/dL), and 9-*cis*-octadecanoic acid (81.7 mg/dL). A study by Barroso et al. [80] also found an analogous relationship in which triglyceride levels increased by about 10% with a high-fat diet (393.5 mg/dL) and with a high-fat diet supplemented with *I. paraguariensis* extract (416.7 mg/dL). However, although mate prevented the accumulation of fatty droplets inside hepatocytes, the extract was not able to counteract the increase in serum triglycerides. In de Morais’ study [96] in dyslipidemic organisms, green and roasted Yerba mate extract in rats had no significant effects on triglyceride levels. The author suggested, however, that longer studies should be conducted to investigate the effect of *I. paraguariensis* on atherosclerotic plaques in humans. Comparatively, a study conducted by Gao [97] showed different results in hamsters, in which one group had hyperlipidemia and was fed a high-fat diet and an analogous one with the addition of Yerba mate extract; the latter group showed a 36% reduction in total cholesterol and a decrease in triglycerides (4.9 mmol/L in the control group vs. 3.12 mmol/L in the test group). The beneficial effects of mate may have resulted from the reduction of lipid peroxidation, increased lipolysis, and the effects on genes responsible for lipid metabolic processes. A completely different outcome was presented by Arcari et al. [93] in their study. A 44% increase in triglycerides was found in control groups. In that study, one group was fed a standard diet and the other a high-fat diet, but a 33% decrease in triglycerides was found in an experimental group supplemented with mate extract. This suggested that mate extracts in vivo had a strong anti-obesity effect and a modulating effect on the expression of genes responsible for the obesity process.

#### 3.7.3. Urine

The analysis of fatty acid esters in urine samples revealed 25 compounds, of which the five main ones are shown in Table 12 (a table containing all compounds is available in the Supplementary Materials). The main components were octadecanoic acid (42.28–30.94 mg/dL) and hexadecanoic acid (26.59–7.76 mg/dL). A 14% reduction in triglycerides was observed in the ILEX group, as compared to the control group. No change in triglycerides was observed in the TERP group, whereas an 11% increase in triglycerides was found in the TERPCHOL group.

In summary, the results presented indicate that the only group in which a reduction in the total triglyceride levels in both the plasma and liver was observed was the *Ilex* water macerate group. An increase of more than 20% in liver triglycerides was observed on the high-cholesterol diet, with similar levels in plasma. In contrast, the TERPCHOL fraction increased urinary TG levels in a statistically significant manner. A similar increase in the total plasma TG concentration was observed by Balzan et al. [98] after the addition of extracts (70% EtOH) of *I. paraguariensis*. This indicates a fundamental change in the metabolism of the lipid metabolism process. De Morais [96] similarly observed a 12% increase in plasma triglyceride levels after 20 days of Yerba mate administration.

### 3.8. Histopathological Examination of Tissue

**Figure 5:**
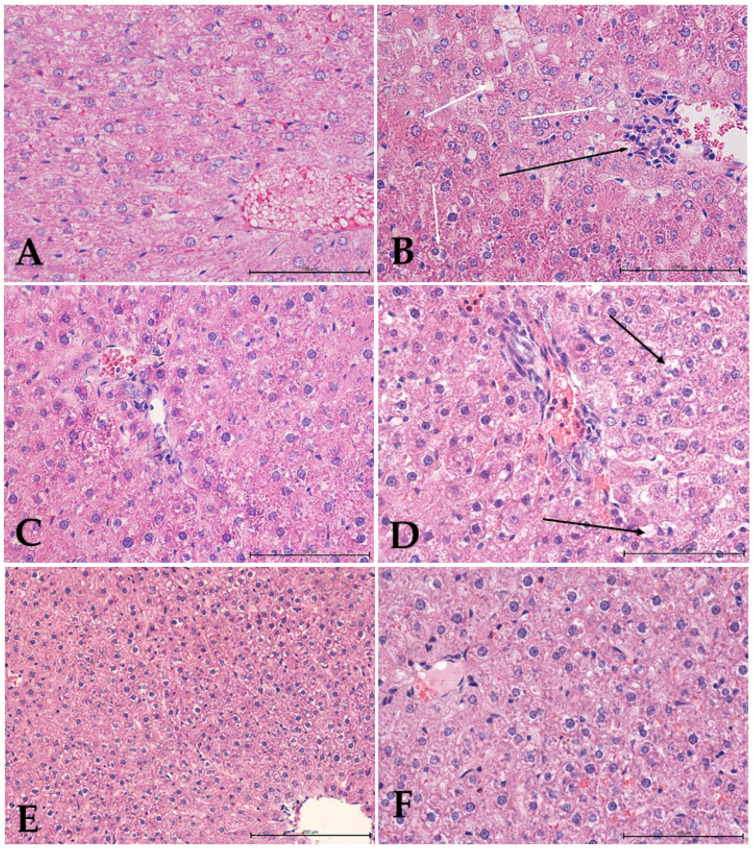
Histological examination of rat livers stained with hematoxylin and eosin (H&E). (**A**) Control group, showing liver tissue with normal architecture; (**B**) high-cholesterol group, showing hepatocytes with intracytoplasmic fat vacuoles (white arrows), small lymphatic infiltration around the liver vessels (black arrow) and numerous binuclear hepatocytes; (**C**) *Ilex* group, showing liver tissue with normal architecture; (**D**) terpenoids group, showing numerous hepatocytes with changes within the cytoplasm (black arrows); (**E**,**F**) high-cholesterol with terpenoids group, showing normal liver structure with increased content of fat droplets in hepatocytes. Mag. 400×. Bar scale 100 µm (**A**–**D**,**F**); Mag. 200×. bar scale 200 µm (**E**).

The liver is the most important organ, and it predominantly controls the metabolism of endogenous and exogenous agents, as well as glucose and lipid metabolisms. As a result, many substances can react with the basic cellular components and, consequently, induce many types of liver damage and dysfunction [99,100]. In our research, the histological investigation of the rat livers (Figure 5) in the control group showed a normal liver architecture, with hepatocytes containing a single, centrally located nucleus. The hepatocytes were characterized by individual cytoplasmic fat vacuoles with no sign of inflammation, necrosis, or cholestasis. In the groups of rats fed a high-cholesterol diet, the histological examinations of the liver sections demonstrated steatosis and minor lymphocytic infiltration around the liver vessels and enlarged portal-bile spaces. Some of the hepatocytes showed changes in cell nuclei, including pyknotic nuclei, a reduced form of the nucleolus, and the presence of more heterochromatin. The presence of numerous binuclear hepatocytes may indicate an intensifying process of cell proliferation in response to damage. The liver structures of the ILEX rats demonstrated an enlargement of the blood vessels, mainly the central veins, with no signs of increased cellular blood components within the sinusoids or the normal portal bile spaces or of steatosis changes in hepatocytes due to increased lipid storage. In rats supplemented with terpenoid fractions, the hepatocytes featured swelling and blood stagnation in both arterial and venous vessels, with no perivascular infiltration visible. The architecture of the lobuli was disturbed. The sinusoids were poorly visible in contrast to the portal-bile spaces. Numerous hepatocyte changes within the cytoplasm were observed, including an increased volume of cells and/or pycnotic nuclei and the cytoplasm itself was translucent, without any granules or solid material. In the livers of the TERPCHOL group, small perivascular lymphocytic infiltrates were present. The sinusoids were clearly visible, whereas the biliary canaliculi were poorly visible. The structures of the cell nuclei and the cytoplasm were similar to the TERP group, except for the increase in the content of fat and water vacuoles. The level of liver damage and the number of bi-nucleated or multinucleated hepatocytes decreased, suggesting that the overall damage had been lessened.

*Ilex* fractions show the potential for liver protection [101,102], and the consumption of the *Ilex* extract may be used as a preventative step for hypercholesterolemia [97,103]. Similarly, terpenoids have been shown to have a protective effect in the liver and may have potential in the treatment of liver diseases [104]. Terpenoids have been applied as activators of peroxisome proliferator-activated receptors (PPAR) and may decrease the risk of obesity-associated disorders [97,105,106]. In our study, histopathological assessments of the liver sections from the control group showed normal hepatic tissues, whereas steatosis was observed in animals fed a high-cholesterol diet. Cholesterol and LDL levels, as well as AST and ALT levels, are important indicators of liver function, and we found that AST and ALT levels were elevated in rats fed a high-cholesterol diet. Histologically, we observed liver steatosis and small leukocyte infiltrates in these animals, which indicated metabolic disruption, as compared to the control group. The inflammatory infiltrates were visible in every group receiving the high-cholesterol diet. In this study, *Ilex* extracts reduced the fatty liver changes in animals fed a high-cholesterol diet. This affirmed the results of previous studies, in which *Ilex* extracts were shown to decrease the differentiation of pre-adipocytes and to reduce the accumulation of lipids in adipocytes and overall inflammation, as well as suppressing adipocyte differentiation and triglyceride accumulation [102]. As compared to other studies [103,104], the animals treated with terpenoids showed prominent changes that are typical of liver damage, including higher rates of hepatocyte swelling and hepatocyte vacuolization, as well as the shifting of nuclei to the cellular periphery and cytoplasmic vacuolization. In addition, our research did not show that proliferative processes were intensified, which may indicate that terpenoids have an inhibitory effect on the proliferation of hepatocytes.

## 4. Conclusions

Our study showed that the terpenoid profiles of the *Paraguariensis* and *Aquifoilum* species are similar. The predominant components were triterpene ursolic acids and both isomers of amyrins. It was shown that the aqueous fractions, despite the lack of caffeine, exhibited a strong hypocholesterolemic effect. This contradicts previous reports that the hypolipidemic effect of Yerba mate (*I. paraguariensis*) is caused solely by the presence of caffeine. Our in vivo study showed that *I. aquifolium* and its terpenoids had an impact on serum lipid levels in an animal model of hyperlipidemia. *I. aquifolium* improved insulin sensitivity and decreased the blood concentrations of total cholesterol and LDL. Supplementation with terpenoids may be an effective way to reduce oxidative damage to lipids, as it decreases the concentration of MDA and leads to an increase in blood TAS. However, terpenoids also appeared to mitigate liver damage. *I. aquifolium* extract reduced the fatty liver changes in animals fed a high-cholesterol diet. Therefore, these results indicate that the aqueous extract of *I. aquifolium* may be an alternative treatment for hyperlipidemia. The limitations of this research were that not all genes regulating lipid metabolism showed significant improvements after being treated with the terpenoids. This will be examined in our future research.

## Figures and Tables

**Table 1:** Multiple reaction monitoring (MRM) transitions and mass spectrometric conditions used for the identification of caffeine and theobromine isolated from *I. paraguariensis* and various cultivars of *I. aquifolium*.

Compound	MRM Transition *m*/*z* (Q1 → Q3)	Q1 (V)	CE (V)	Q3 (V)
Caffeine	195.2 → 138.10	−10.0	−22	−22.0
	195.2 → 42.15	−10.0	−36	−15.0
Theobromine	181.1 → 138.10	−10.0	−22	−22.0
	181.1 → 89.10	−10.0	−31	−18.0

**Table 2:** Nutritional value of pelleted feed fed to Wistar rats.

Ingredient	Content
Dry weight (g/kg)	906.04
Energy (MJ/kg)	19.78
Total protein (% d.w.)	17.42
Crude fat (% d.w.)	2.13
Crude fiber (% d.w.)	9.45
ADF (% d.w.)—Acid detergent fiber fraction	8.42
NDF (% d.w.)—Neutral detergent fiber fraction	18.38
Crude ash (% d.w.)	4.77

**Table 3:** Triterpenoid profile of *I. paraquariensis* and various varieties of *I. aquifolium*.

Compound	RI Exp. ^1^	RI Lit. ^2^	*m*/*z*	*I. paraguariensis*	*I. aquifolium*	*I. aquifolium* Alaska	*I. aquifolium* Ferox Argentea
				Concentration (mg/g) d.w			
β-Sitosterol TMS	3370	3344	129/357/396	0.99 ± 0.11 ^3,4,a^	1.23 ± 0.21 ^c^	0.96 ± 0.20 ^a^	1.09 ± 0.23 ^b^
β-Amyrin TMS	3384	3353	218/203/190	1.15 ± 0.31 ^a^	1.45 ± 0.46 ^b^	1.60 ± 0.31 ^c^	1.63 ± 0.41 ^c^
α-Amyrin TMS	3420	3406	218/189/73	3.85 ± 0.87 ^a^	4.64 ± 0.89 ^c^	4.47 ± 0.19	4.89 ± 0.37 ^d^
Lupeol TMS	3427	3435	189/109/190	1.20 ± 0.43 ^a^	2.77 ± 0.84 ^d^	2.52 ± 0.09 ^c^	2.04 ± 0.31 ^b^
Uvaol 2O-TMS	3530	3540	73/496/203	2.14 ± 0.56 ^a^	0.38 ± 0.09 ^c^	0.47 ± 0.11 ^b^	0.34 ± 0.11 ^c^
Betulinic alcohol 2O-TMS	3563	3560	203/189/73	3.05 ± 0.70 ^a^	1.94 ± 0.67 ^c^	2.19 ± 0.76 ^b^	1.49 ± 0.26 ^d^
Betulinic acid 2O-TMS	3580	3588	73/189/129	1.16 ± 0.61 ^a^	0.30 ± 0.10 ^b^	0.24 ± 0.05 ^c^	0.20 ± 0.09 ^c^
Oleanolic acid TMS	3596	3591	203/73/189	1.81 ± 0.34 ^a^	0.16 ± 0.03 ^b^	0.10 ± 0.01 ^c^	0.17 ± 0.04 ^b^
Ursolic acid TMS	3643	3657	203/73/320	0.64 ± 0.09 ^a^	0.84 ± 0.16 ^d^	0.43 ± 0.08 ^c^	0.54 ± 0.11 ^b^
Ursolic aldehyde TMS	3668	3672	203/190/133	0.40 ± 0.05 ^a^	1.05 ± 0.13 ^d^	0.75 ± 0.13 ^b^	0.90 ± 0.21 ^c^
TOTAL				16.39 ^a^	14.75 ^b^	13.72 ^c^	13.30 ^c^

^1^ Experimental retention indices calculated against n-alkanes. ^2^ Retention indices according to the NIST 20 database. ^3^ Values are mean ± SEM; values followed by the same letter within a row are not significantly different (*p* > 0.05, Duncan’s test). ^4^ Data are relative concentrations expressed per internal standard.

**Table 4:** Phenolic acid profile of *I. paraquariensis* and various varieties of *I. aquifolium*.

Compound	RI Exp. ^1^	RI Lit. ^2^	*m*/*z*	*I. paraguariensis*	*I. aquifolium*	*I. aquifolium* Alaska	*I. aquifolium* Ferox Argentea
				Concentration (mg/g) d.w.			
Malic acid, 3TMS derivative	1502	1497	73/147/233	0.07 ± 0.02 ^3,4,a^	0.55 ± 0.13 ^c^	0.26 ± 0.09 ^b^	0.26 ± 0.11 ^b^
Citric acid, 4TMS derivative	1856	1845	273/73/147	0.16 ± 0.12 ^a^	0.06 ± 0.02 ^c^	0.11 ± 0.04 ^b^	0.11 ± 0.05 ^b^
Quininic acid, 5TMS derivative	1863	1851	373/374/377	0.01 ± 0.01 ^a^	0.19 ± 0.07 ^b^	0.22 ± 0.07 ^c^	0.22 ± 0.11 ^c^
Ferulic acid, 2TMS derivative	2102	2104	338/73/323	0.20 ± 0.08 ^a^	0.11 ± 0.05 ^b^	0.06 ± 0.02 ^c^	0.06 ± 0.03 ^c^
Caffeic acid, 3TMS derivative	2163	2155	396/219/73	2.43 ± 0.27 ^a^	2.88 ± 0.43 ^c^	1.68 ± 0.31 ^b^	1.64 ± 0.24 ^b^
3-O-Coumaroyl-D-quinic acid, 5TMS derivative	3043	3048	219/345/73	1.03 ± 0.09 ^a^	0.07 ± 0.04 ^b^	0.03 ± 0.02 ^c^	0.04 ± 0.01 ^c^
Chlorogenic acid, 6TMS derivative	3081	3092	345/73/255	1.08 ± 0.18 ^a^	0.05 ± 0.02 ^b^	0.03 ± 0.01 ^c^	0.03 ± 0.01 ^c^
4-O-Caffeoylquinic acid, 6TMS derivative	3096	3095	219/73/255	0.80 ± 0.13 ^a^	0.04 ± 0.03 ^b^	0.02 ± 0.01 ^c^	0.02 ± 0.01 ^c^
TOTAL				5.76 ^a^	3.93 ^b^	2.40 ^c^	2.38 ^c^

^1^ Experimental retention indices calculated against n-alkanes. ^2^ Retention indices according to the NIST 20 database. ^3^ Values are mean ± SEM; values followed by the same letter within a row are not significantly different (*p* > 0.05, Duncan’s test). ^4^ Data are relative concentrations expressed per internal standard.

**Table 5:** Sugar profile of *I. paraquariensis* and various varieties of *I. aquifolium*.

Compound	RI Exp. ^1^	RI Lit. ^2^	*m*/*z*	*I. paraguariensis*	*I. aquifolium*	*I. aquifolium* Alaska	*I. aquifolium* Ferox Argentea
				Concentration (mg/g) d.w.			
Fructose (TMS)	1922	1867	73/217/204	0.95 ± 0.13 ^3,4,a^	0.63 ± 0.14 ^b^	0.64 ± 0.12 ^b^	0.37 ± 0.09^c^
Mannose isomer 1 (TMS)	1933	1944	205/103/160	0.73 ± 0.11 ^a^	0.49 ± 0.12 ^b^	0.45 ± 0.09 ^b^	0.25 ± 0.08 ^c^
Glucose isomer 1(TMS)	1948	1964	204/73/205	1.77 ± 0.21 ^a^	0.89 ± 0.23 ^b^	0.92 ± 0.11 ^b^	0.50 ± 0.05 ^c^
Glucose isomer 2 (TMS)	1963	1980	204/73/205	0.45 ± 0.29 ^a^	0.18 ± 0.08 ^b^	0.18 ± 0.03 ^b^	0.11 ± 0.01 ^c^
Mannose isomer 2 (TMS)	2499	2561	205/103/160	0.37 ± 0.22 ^a^	0.37 ± 0.11 ^a^	0.36 ± 0.06 ^a^	0.21 ± 0.02 ^b^
Sucrose (TMS)	2580	2598	361/73/362	2.05 ± 0.33 ^a^	2.80 ± 0.24^c^	2.93 ± 0.21^d^	1.58 ± 0.12 ^b^
D-Cellobiose isomer 1 (TMS)	2641	2631	204/191/217	-	1.13 ± 0.17	1.19 ± 0.17	0.60 ± 0.24
Maltose isomer 1 (TMS)	2656	2657	204/73/191	-	0.29 ± 0.07	0.30 ± 0.09	0.17 ± 0.06
D-Cellobiose isomer 1 (TMS)	2695	2693	204/191/217	-	0.25 ± 0.05	0.26 ± 0.03	0.14 ± 0.07
Maltose isomer 2 (TMS)	2711	2712	204/73/191	-	0.95 ± 0.10	0.96 ± 0.13	0.54 ± 0.12
Gentiobiose	2916	2964	361/190/217	0.58 ± 0.25 ^a^	0.60 ± 0.12^a^	0.60 ± 0.12^d^	0.34 ± 0.09 ^b^
TOTAL				6.91 ^a^	8.57 ^b^	8.78 ^b^	4.82 ^c^

^1^ Experimental retention indices calculated against n-alkanes. ^2^ Retention indices according to the NIST 20 database. ^3^ Values are mean ± SEM; values followed by the same letter within a row are not significantly different (*p* > 0.05, Duncan’s test). ^4^ Data are relative concentrations expressed per internal standard.

**Table 6:** Comparison of the quantity of caffeine and theobromine isolated from *I. paraguariensis* and various *I. aquifolium* cultivars analyzed by means of GC-MS and LC-MS.

	**GC-MS**				**LC-MS**			
	**Caffeine**		**Theobromine**		**Caffeine**		**Theobromine**	
	**LOD**	**LOQ**	**LOD**	**LOQ**	**LOD**	**LOQ**	**LOD**	**LOQ**
	(ng/mL)							
	25.12	54.87	36.91	64.23	4.01	12.34	5.37	15.21
	Concentration (mg/g) d.w.							
*I. paraguariensis*	6.21 ± 0.54 ^1^		1.01 ± 0.32		7.77 ± 0.26		1.38 ± 0.19	
*I. aquifolium*	below detection level				below detection level			
*I. aquifolium* Alaska	below detection level				below detection level			
*I. aquifolium* Ferox Argentea	below detection level				below detection level			

^1^ Values are mean ± SEM.

**Table 7:** Hematological parameters in normal or hypercholesterolemic Wistar rats after treatment with *I. aquifolium* and terpenoid fractions.

	Group					*p* < Value
	CON	CHOL	ILEX	TERP	TERPCHOL	
WBC ^2^ G/L	6.06 ± 1.38 ^a,1^	5.03 ± 0.67 ^b^	7.26 ± 2.32 ^b^	5.81 ± 1.48 ^b^	8.30 ± 1.43 ^b^	<0.001
RBC ^3^ T/L	7.63 ± 0.58 ^a^	8.58 ± 0.35 ^b,c^	8.18 ± 1.16 ^b,c^	7.35 ± 0.84 ^b^	9.72 ± 0.43 ^b,c^	<0.001
HGB ^4^ mmol/L	9.23 ± 0.71 ^a^	9.43 ± 0.25 ^a^	10.04 ± 1.42	8.90 ± 0.97 ^a^	11.06 ± 0.64 ^b^	<0.001
HCT ^5^ L/L	0.41 ± 0.04 ^a^	0.45 ± 0.01 ^a^	0.45 ± 0.06 ^a^	0.39 ± 0.05 ^a^	0.52 ± 0.03 ^b^	0.019
MCV ^6^ fL	53.56 ± 1.59 ^a^	52.86 ± 1.07	55.13 ± 0.83 ^b^	53.13 ± 0.99 ^a^	53.13 ± 1.89	<0.001
MCH ^7^ fmoL	1.21 ± 0.06 ^a^	1.10 ± 0.03 ^c^	1.23 ± 0.03 ^c^	1.21 ± 0.02 ^c^	1.14 ± 0.03b ^c^	<0.001
MCHC ^8^ mmol/L	22.57 ± 0.63 ^a^	20.87 ± 0.24 ^c^	22.28 ± 0.40 ^c^	22.70 ± 0.32 ^c^	21.48 ± 0.48 ^b,c^	<0.001
PLT ^9^ G/L	592.11 ± 65.98	615.57 ± 92.54	768.25 ± 66.07	693.50 ± 96.67	552.38 ± 79.09	0.039

^1^ Values are mean ± SEM; different letters within a row indicate significant differences between groups (*p* < 0.05; ANOVA followed by the post-hoc test); ^2^ white blood cells; ^3^ red blood cells; ^4^ hemoglobin; ^5^ hematocrit; ^6^ mean corpuscular volume; ^7^ mean corpuscular hemoglobin; ^8^ mean corpuscular hemoglobin concentration; ^9^ platelets.

**Table 8:** Mean values of lipid and biochemical parameters and activity of selected enzymes in normal or hypercholesterolemic Wistar rats.

	Group					*p* < Value
	CON	CHOL	ILEX	TERP	TERPCHOL	
Chol. ^2^ mmol/L	1.57 ± 0.18 ^1,a,c^	2.41 ± 0.40 ^b^	1.31 ± 0.23 ^c^	1.62 ± 0.17 ^c^	2.07 ± 0.35 ^b^	<0.001
HDL ^3^ mmol/L	0.72 ± 0.07	0.83 ± 0.09	0.70 ± 0.20	0.74 ± 0.10	0.83 ± 0.08	0.082
LDL ^4^ mmol/L	0.85 ± 0.08 ^a^	1.58 ± 0.03 ^b^	0.61 ± 0.09 ^c^	0.88 ± 0.07	1.24 ± 0.11 ^b^	<0.001
TG ^5^ mmol/L	1.07 ± 0.24 ^a,c^	1.62 ± 0.22 ^b^	0.94 ± 0.32 ^c^	1.22 ± 0.27 ^c^	2.01 ± 0.71 ^b^	<0.001
NEFA ^6^ mmol/L	0.48 ± 0.25	2.02 ± 0.24	0.38 ± 0.16	0.57 ± 0.18	1.65 ± 0.67	0.068
Glucose mmol/L	4.75 ± 0.77 ^a^	7.58 ± 1.13 ^b^	5.31 ± 0.97 ^a^	5.16 ± 1.21 ^a^	6.02 ± 1.12 ^a^	<0.001
Insulin pg/mL	56.67 ± 54.87 ^a^	201.33 ± 113.65 ^b^	84.71 ± 7.34 ^a^	85.14 ± 31.71 ^a^	68.83 ± 16.07 ^a^	<0.001
TP ^7^	73.71 ± 11.73 ^a^	65.45 ± 3.17	62.75 ± 3.78 ^b^	64.95 ± 3.02 ^b^	66.01 ± 2.41	0.010
Alb. ^8^	34.28 ± 3.66 ^a^	30.53 ± 1.38 ^b^	29.85 ± 1.35 ^b^	30.59 ± 1.55 ^b^	31.67 ± 0.73	<0.001
AST ^9^ U/L	143.17 ± 33.45 ^a,c^	221.88 ± 55.23 ^b^	210.84 ± 57.53 ^b,c^	188.96 ± 44.45	146.57 ± 22.67 ^c^	0.002
ALT ^10^ U/L	77.76 ± 5.35	93.56 ± 12.64	89.88 ± 28.64	80.29 ± 16.12	71.09 ± 9.58	0.071
Creatinine	43.69 ± 10.78	50.70 ± 7.78	54.03 ± 15.17 ^a^	53.39 ± 6.77 ^a^	37.29 ± 3.66 ^b^	0.009

^1^ Values are mean ± SEM; different letters within a row indicate significant differences between groups (*p* < 0.05; ANOVA followed by the post-hoc test); ^2^ cholesterol; ^3^ high-density lipoprotein; ^4^ low-density lipoprotein; ^5^ triglycerides; ^6^ non-estrified fatty acids; ^7^ total protein; ^8^ albumin; ^9^ aspartate aminotransferase; ^10^ alanine transaminase.

**Table 9:** Effects of *I. aquifolium* and terpenoids on immunological and antioxidant parameters in normal or hyperlipidemic Wistar rats.

	Group					*p* < Value
	CON	CHOL	ILEX	TERP	TERPCHOL	
IgG ^2^ g/L	11.20 ± 2.64 ^a,1^	14.01 ± 3.48	14.07 ± 4.16	12.42 ± 2.95	15.73 ± 4.50 ^b^	0.052
IgM ^3^ mg/mL	0.56 ± 0.19	0.62 ± 0.15	0.73 ± 0.20 ^a^	0.51 ± 0.13 ^b^	0.62 ± 0.12	0.068
IL-6 ^4^ pg/mL	37.49 ± 13.67	39.71 ± 5.28	32.27 ± 5.99	26.99 ± 14.86	34.99 ± 5.08	0.130
IL-10 ^5^ pg/mL	14.11 ± 9.85 ^a^	4.05 ± 3.09 ^b^	14.43 ± 7.34 ^b^	17.58 ± 5.47 ^b^	12.85 ± 4.33 ^b^	<0.001
TAS ^6^ mmol/L	1.27 ± 0.30 ^a,c^	0.97 ± 0.05b ^c^	0.92 ± 0.12 ^b,c^	1.34 ± 0.06 ^b^	1.12 ± 0.10 ^c^	<0.001
MDA ^7^ ng/mL	104.17 ± 47.78 ^a^	468.94 ± 44.40 ^b^	368.28 ± 48.28 ^b^	81.43 ± 16.86 ^c^	419.75 ± 69.52 ^b^	<0.001
GPx ^8^ U/L	136.55 ± 28.96	149.87 ± 38.62	129.69 ± 29.22	131.99 ± 84.14	150.03 ± 23.98	0.061
TOS ^9^ µmol/L	10.27 ± 6.54	8.77 ± 2.02	13.29 ± 3.75	9.28 ± 3.34	10.03 ± 2.79	0.086

^1^ Values are mean ± SEM; different letters within a row indicate significant differences between groups (*p* < 0.05; ANOVA followed by the post-hoc test); ^2^ immunoglobulin G; ^3^ immunoglobulin M; ^4^ interleukin-6; ^5^ interleukin-10; ^6^ total antioxidant status; ^7^ malondialdehyde; ^8^ glutathione peroxidase; ^9^ total oxidant status.

**Table 10:** Fatty acid profile of liver tissue in normal or hypercholesterolemic Wistar rats after treatment with *I. aquifolium* and terpenoid fraction.

RT ^1^	Name	*m*/*z*	Group				
			CON ^4^	CHOL ^5^	ILEX ^6^	TERP ^7^	TERPCHOL ^8^
			Concentration (mg/g)				
29.77	Hexadecanoic acid methyl ester	74/87/99	12.39 ± 2.48 ^2,3,a^	15.17 ± 1.45 ^c^	12.58 ± 2.42 ^b^	16.75 ± 2.05 ^d^	20.21 ± 2.86 ^e^
30.67	9-*cis*-Hexadecenoic acid methyl ester	55/96/87	4.75 ± 0.53 ^a^	6.25 ± 1.06 ^b^	4.67 ± 0.71 ^a^	6.90 ± 0.66 ^c^	8.44 ± 1.12 ^d^
34.55	Octadecanoic acid methyl ester	74/87/55	6.11 ± 0.74 ^a^	8.00 ± 0.76 ^c^	4.82 ± 0.91 ^b^	8.84 ± 0.91 ^d^	9.68 ± 1.05 ^e^
35.23	9-*trans*-Octadecenoic acid methyl ester	264/97/83	8.40 ± 1.89 ^a^	11.99 ± 1.13 ^b^	8.25 ± 1.99 ^a^	13.24 ± 1.13 ^c^	14.91 ± 1.17 ^d^
36.49	Linoleic acid methyl ester	67/81/95	4.44 ± 0.86 ^a^	4.71 ± 0.56 ^b^	3.37 ± 0.99 ^d^	5.20 ± 0.92 ^c^	7.19 ± 0.86 ^e^
40.47	Arachidonic acid methyl ester	79/91/67	6.51 ± 1.33 ^a^	5.97 ± 0.23 ^b^	4.59 ± 1.06 ^d^	6.60 ± 0.99 ^a^	7.73 ± 1.02 ^c^
TOTAL			42.60 ^a^	52.09 ^c^	38.28 ^b^	57.53 ^d^	68.16 ^e^

^1^ Retention time (min). ^2^ Values are mean ± SEM, values followed by the same letter within a row are not significantly different (*p* > 0.05, Duncan’s test). ^3^ Data are relative concentrations expressed per internal standard ^4^ control group, ^5^ group fed a high-cholesterol diet, ^6^ rats fed a standard diet with the addition of an aqueous extract of *I. aquifolium,* ^7^ animals fed a standard diet with the addition of terpenoid fractions, and ^8^ rats fed a high-cholesterol diet with the addition of terpenoid fractions.

**Table 11:** Fatty acid profile of blood serum in normal or hypercholesterolemic Wistar rats after treatment with *I. aquifolium* and terpenoid fraction.

RT ^1^	Name	*m*/*z*	Group				
			CON ^4^	CHOL ^5^	ILEX ^6^	TERP ^7^	TERPCHOL ^8^
			Concentration (mg/dL)				
29.36	Hexadecanoic acid methyl ester	74/87/99	20.17 ± 2.04 ^2,3,a^	18.67 ± 0.27 ^b^	7.60 ± 0.14 ^e^	27.43 ± 0.61 ^d^	26.37 ± 4.87 ^c^
34.14	Octadecanoic acid methyl ester	55/96/87	23.59 ± 2.27 ^a^	26.57 ± 1.60 ^c^	8.81 ± 0.11 ^d^	22.49 ± 0.14 ^b^	26.38 ± 4.53 ^c^
34.82	9-*cis*-Octadecenoic acid methyl ester	264/97/83	9.41 ± 0.07 ^a^	12.25 ± 1.23 ^b^	4.55 ± 0.11 ^c^	4.72 ± 0.17 ^c^	17.93 ± 2.38 ^d^
36.22	Linoleic acid methyl ester	74/87/55	8.14 ± 0.57 ^a^	9.31 ± 0.28 ^b^	2.86 ± 0.06 ^d^	9.30 ± 0.58 ^b^	13.20 ± 1.75 ^c^
TOTAL			61.31 ^a^	66.81 ^c^	23.82 ^e^	63.94 ^b^	83.88 ^d^

^1^ Retention time. ^2^ Values are mean ± SEM; values followed by the same letter within a row are not significantly different (*p* > 0.05, Duncan’s test). ^3^ Data are relative concentrations expressed per internal standard ^4^ control group, ^5^ group fed a high-cholesterol diet, ^6^ rats fed a standard diet with the addition of an aqueous extract of *I. aquifolium,* ^7^ animals fed a standard diet with the addition of terpenoid fractions, and ^8^ rats fed a high-cholesterol diet with the addition of terpenoid fractions.

**Table 12:** Fatty acid profile of urine in normal or hypercholesterolemic Wistar rats after treatment with *I. aquifolium* and terpenoid fraction.

RT ^1^	Name	*m*/*z*	Group				
			CON ^4^	CHOL ^5^	ILEX ^6^	TERP ^7^	TERPCHOL ^8^
			Concentration (mg/dL)				
29.33	Hexadecanoic acid methyl ester	74/87/99	7.76 ± 1.99 ^2,3,a^	11.76 ± 2.53 ^d^	6.74 ± 1.34 ^b^	10.58 ± 2.01 ^c^	26.59 ± 3.01 ^e^
34.11	Octadecanoic acid methyl ester	55/96/87	30.94 ± 3.21 ^a^	28.37 ± 4.16 ^b^	26.07 ± 3.99 ^c^	26.24 ± 3.12 ^c^	42.28 ± 2.76 ^d^
34.79	9-*cis*-Octadecenoic acid methyl ester	264/97/83	0.71 ± 0.13 ^a^	1.55 ± 0.82 ^d^	0.66 ± 0.07 ^b^	1.06 ± 0.09 ^c^	3.59 ± 1.16 ^e^
34.98	9-*trans*-Octadecenoic acid methyl ester	264/97/83	0.62 ± 0.15 ^a^	1.87 ± 0.22 ^d^	0.71 ± 0.11 ^b^	1.54 ± 0.13 ^c^	4.16 ± 1.01 ^e^
42.34	11-*trans*-Eicosenoic acid methyl ester	41/55/69	0.78 ± 0.21 ^a^	2.88 ± 0.34 ^c^	0.80 ± 0.06 ^b^	2.94 ± 0.44 ^c^	4.05 ± 0.38 ^d^
TOTAL			40.80 ^a^	46.42 ^c^	34.97 ^d^	42.36 ^b^	80.66 ^e^

^1^ Retention time. ^2^ Values are mean ± SEM; values followed by the same letter within a row are not significantly different (*p* > 0.05, Duncan’s test). ^3^ Data are relative concentrations expressed per internal standard ^4^ control group, ^5^ group fed a high-cholesterol diet, ^6^ rats fed a standard diet with the addition of an aqueous extract of *I. aquifolium*, ^7^ animals fed a standard diet with the addition of terpenoid fractions, and ^8^ rats fed a high-cholesterol diet with the addition of terpenoid fractions.

## Data Availability

The data presented in this study are available in Supplementary Materials www.mdpi.com/article/10.3390/foods11010047/s1.

## Supplementary Materials

The following are available online at https://www.mdpi.com/article/10.3390/foods11010047/s1, Table S1: Triterpenoid profile of *I. paraquariensis* and various varieties of *I. aquifolium.* Table S2: Phenolic acid profile of *I. paraquariensis* and various varieties of *I. aquifolium*. Table S3: Fatty acid profile of liver tissue in normal or hypercholesterolemic Wistar rats after treatment with *I. aquifolium* and terpenoid fraction. Table S4: Fatty acid profile of blood serum in normal or hypercholesterolemic Wistar rats after treatment *I. aquifolium* and terpenoid fraction. Table S5: Fatty acid profile of urine in normal or hypercholesterolemic Wistar rats after treatment *I. aquifolium* and terpenoid fraction.
