# Supplementary material for: Biological Potential and Chemical Profile of European Varieties of Ilex
Source: Foods. 2021 Dec 25;11(1):47. doi: 10.3390/foods11010047 (PMC8750822; doi:10.3390/foods11010047)
Supplement: Supplementary file 1 [file foods-11-00047-s001.zip › foods-1513346-supplementary.pdf]

Table S1: Triterpenoids profile of *Ilex paraquariensis* and various varieties of *Ilex aquifolium*.

**Compound**	**RI exp.**[1]	**RI lit.**[2]	***Ilex paraguariensis***	***Ilex aquifolium***	***Ilex aquifolium* Alaska**	***Ilex aquifolium* Ferox Argentea**
			**Concentration [mg/g] d.w**			
all-trans-Squalene	2835	2832	0.17±0.06	0.10±0.03	0.09±0.02	0.16±0.05
(3β)-Olean-18-en-3-ol, O-TMS	3362	3370	0.08±0.02	0.35±0.12	0.20±0.06	0.09±0.03
β-Sitosterol TMS	3370	3344	0.99±0.11	1.23±0.21	0.96±0.20	1.09±0.23
β-Amyrin TMS	3384	3353	1.15±0.31	1.45±0.46	1.60±0.31	1.63±0.41
Germanicol TMS	3397	3385	0.19±0.05	0.43±0.15	0.31±0.13	0.35±0.11
α-Amyrin TMS	3420	3406	3.85±0.87	4.64±0.89	4.47±0.19	4.89±0.37
Lupeol TMS	3427	3435	1.20±0.43	2.77±0.84	2.52±0.09	2.04±0.31
Lupenyl acetate	3518	3523	1.17±0.15	0.12±0.06	0.17±0.04	0.19±0.08
Uvaol 2O-TMS	3530	3540	2.14±0.56	0.38±0.09	0.47±0.11	0.34±0.11
Betulinic alcohol 2O-TMS	3563	3560	3.05±0.70	1.94±0.67	2.19±0.76	1.49±0.26
Betulinic acid 2O-TMS	3580	3588	1.16±0.61	0.30±0.10	0.24±0.05	0.20±0.09
Oleanolic acid TMS	3596	3591	1.81±0.34	0.16±0.03	0.10±0.01	0.17±0.04
Ursolic acid TMS	3643	3657	0.64±0.09	0.84±0.16	0.43±0.08	0.54±0.11
Ursolic aldehyde TMS	3668	3672	0.40±0.05	1.05±0.13	0.75±0.13	0.90±0.21

[1] Experimental retention indices calculated against n-alkanes [2] Retention indices according to NIST 20 database

Table S2: Phenolic acids profile of *Ilex paraquariensis* and various varieties of *Ilex aquifolium*.

**Compound**	**RI exp.**[1]	**RI lit.**[2]	***Ilex paraguariensis***	***Ilex aquifolium***	***Ilex aquifolium* Alaska**	***Ilex aquifolium* Ferox Argentea**
			**Concentration [mg/g] d.w**			
Sulfuric acid, 2TMS derivative	1170	1169	0.08±0.02	0.02±0.01	0.04±0.02	0.04±0.02
Succinic acid, 2TMS derivative	1323	1321	0.08±0.03	0.02±0.01	0.02±0.01	0.02±0.01
Fumaric acid, 2TMS derivative	1357	1353	0.01±0.01	0.03±0.02	0.02±0.01	0.02±0.01
Malic acid, 3TMS derivative	1502	1497	0.07±0.02	0.55±0.13	0.26±0.09	0.26±0.11
Pimelic acid, 2TMS derivative	1617	1610	0.01±0.01	0.01±0.00	0.01±0.00	0.01±0.01
3,4-Dihydroxybenzaldehyde, 2TMS derivative	1628	1612	0.03±0.02	0.01±0.01	0.01±0.01	0.01±0.01
4-Hydroxybenzoic acid, 2TMS derivative	1636	1635	0.02±0.01	0.01±0.01	0.01±0.01	0.01±0.01
Suberic acid, 2TMS derivative	1711	1707	0.05±0.02	0.01±0.01	0.01±0.01	0.01±0.01
Isovanillic acid, 2TMS	1765	1769	0.01±0.01	0.01±0.00	0.01±0.01	0.01±0.00
Azelaic acid, 2TMS derivative	1811	1806	0.18±0.06	0.03±0.01	0.01±0.01	0.02±0.01
Caffeine	1839	1835	0.34±0.11	-	-	-
Citric acid, 4TMS derivative	1856	1845	0.16±0.12	0.06±0.02	0.11±0.04	0.11±0.05
Quininic acid, 5TMS derivative	1963	1851	0.01±0.01	0.19±0.07	0.22±0.07	0.22±0.11
Sebacic acid, 2TMS derivative	1907	1904	0.01±0.01	0.01±0.01	0.01±0.01	0.01±0.01
4-Coumaric acid, 2TMS derivative	1946	1949	0.05±0.02	0.02±0.01	0.03±0.02	0.03±0.02
Methyl caffeate, 2TMS derivative	2000	2002	0.04±0.03	0.07±0.03	0.04±0.02	0.04±0.02
Methyl cis-caffeate, 2TMS derivative	2017	2014	0.03±0.02	0.01±0.01	0.01±0.01	0.01±0.01
Palmitic Acid, TMS derivative	2057	2050	0.16±0.07	0.03±0.01	0.02±0.01	0.02±0.01
Isoferulic acid, 2TMS derivative	2082	2079	0.03±0.02	0.05±0.02	0.01±0.00	0.01±0.01
Ferulic acid, 2TMS derivative	2102	2104	0.20±0.08	0.11±0.05	0.06±0.02	0.06±0.03
Caffeic acid, 3TMS derivative	2163	2155	2.43±0.27	2.88±0.43	1.68±0.31	1.64±0.24
Phytol, TMS derivative	2187	2180	0.02±0.01	0.01±0.01	0.01±0.00	0.01±0.01
Sinapinic acid, 2TMS derivative	2259	2267	0.03±0.02	0.03±0.01	0.02±0.01	0.01±0.01
3-O-Coumaroyl-D-quinic acid, 5TMS derivative	3043	3048	1.03±0.09	0.07±0.04	0.03±0.02	0.04±0.01
5-O-Feruloylquinic acid, 5TMS	3065	3074	0.14±0.06	0.03±0.01	0.01±0.01	0.01±0.01
Chlorogenic acid, 6TMS derivative	3081	3092	1.08±0.18	0.05±0.02	0.03±0.01	0.03±0.01

4-O-Caffeoylquinic acid, 6TMS derivative	3096	3095	0.80±0.13	0.04±0.03	0.02±0.01	0.02±0.01
Quercetin, 5TMS derivative	3189	3173	0.16±0.05	0.02±0.01	0.01±0.00	0.01±0.01
Tocopherol	3260	3269	0.16±0.07	0.05±0.03	0.01±0.01	0.01±0.01

[1] Experimental retention indices calculated against n-alkanes [2] Retention indices according to NIST 20 database

Table S3: Fatty acid profile of liver tissue in normal or hypercholesterolemic Wistar rats after treatment *Ilex aquifolium* and terpenoid fraction.

RT	Name	Group				
		**CON**	**CHOL**	***I. aquifolium***	**TERP**	**TERPCHOL**
		**Concentration [g/100g]**				
10.75	Nonanoic acid, methyl ester	0.01±0.01	0.01±0.00	0.01±0.01	0.01±0.00	0.01±0.00
13.35	Decanoic acid, methyl ester	0.01±0.00	0.01±0.01	0.01±0.01	0.01±0.01	0.01±0.01
18.93	Dodecanoic acid, methyl ester	0.04±0.02	0.05±0.02	0.04±0.02	0.05±0.02	0.07±0.02
21.70	Tridecanoic acid, methyl ester	0.01±0.00	0.01±0.01	0.01±0.01	0.01±0.00	0.01±0.01
24.44	Tetradecanoic acid, methyl ester	1.13±0.27	1.49±0.34	0.98±0.21	1.65±0.37	1.98±0.48
25.48	9-(*E*)-Tetradecenoic acid, methyl ester	0.02±0.01	0.02±0.01	0.01±0.01	0.02±0.01	0.03±0.01
25.86	9-(*Z*)-Tetradecenoic acid, methyl ester	0.13±0.07	0.17±0.08	0.11±0.04	0.19±0.06	0.25±0.08
26.25	Tetradecanoic acid, 12-methyl-, methyl ester	0.01±0.00	0.01±0.01	0.01±0.01	0.01±0.01	0.01±0.01
27.06	Pentadecanoic acid, methyl ester	0.12±0.06	0.22±0.13	0.08±0.02	0.24±0.09	0.26±0.09
28.40	10-Pentadecenoic acid, methyl ester	0.01±0.01	0.03±0.01	0.01±0.00	0.03±0.01	0.03±0.01
29.77	Hexadecanoic acid, methyl ester	12.39±2.48	15.17±1.45	12.58±2.42	16.75±2.05	20.20±2.86
30.20	6-(*Z*)-Hexadecenoic acid, methyl ester	0.02±0.01	0.02±0.01	0.01±0.01	0.02±0.01	0.03±0.02
30.42	9-(*E*)-Hexadecenoic acid, methyl ester	0.48±0.13	0.86±0.12	0.33±0.13	0.95±0.22	0.82±0.16
30.67	9-(*Z*)-Hexadecenoic acid, methyl ester	4.75±0.53	6.25±1.06	4.67±0.71	6.90±0.66	8.44±1.12
30.93	11-(*Z*)-Hexadecenoic acid, methyl ester	0.13±0.08	0.16±0.06	0.07±0.02	0.18±0.07	0.25±0.05
31.31	Hexadecanoic acid, 14-methyl-, methyl ester	0.01±0.01	0.02±0.01	0.01±0.01	0.02±0.01	0.03±0.01
32.87	10-(*E*)-Heptadecenoic acid, methyl ester	0.10±0.03	0.22±0.09	0.08±0.03	0.24±0.11	0.27±0.09
33.30	Heptadecanoic acid, 16-methyl-, methyl ester	0.03±0.01	0.03±0.02	0.01±0.01	0.04±0.02	0.06±0.02
34.55	Octadecanoic acid, methyl ester	6.11±0.56	8.00±0.76	4.82±0.91	8.84±0.91	9.68±1.05
35.23	9-(*E*)-Octadecenoic acid, methyl ester	8.40±1.89	11.99±1.13	8.25±1.99	13.24±1.13	14.91±1.17
35.37	9-(*Z*)-Octadecenoic acid, methyl ester	2.21±0.29	3.07±0.46	1.64±0.41	3.39±0.76	3.63±0.59
35.63	11-(*Z*)-Octadecenoic acid, methyl ester	0.07±0.03	0.13±0.05	0.05±0.02	0.14±0.05	0.16±0.08
35.81	7,10-Octadecadienoic acid, methyl ester	0.01±0.01	0.02±0.01	0.01±0.01	0.02±0.01	0.03±0.02
36.11	6,9-Octadecadienoic acid, methyl ester	0.01±0.01	0.04±0.02	0.01±0.00	0.04±0.03	0.04±0.01
36.49	Linoleic acid, methyl ester	4.44±0.86	4.71±0.56	3.36±0.99	5.20±0.92	7.19±0.86
37.15	10-(*Z*)-Nonadecenoic acid, methyl ester	0.01±0.01	0.01±0.00	0.01±0.01	0.01±0.00	0.02±0.01

37.26	γ-Linolenic acid, methyl ester	0.10±0.01	0.16±0.08	0.05±0.02	0.18±0.06	0.22±0.12
37.85	α-Linolenic acid, methyl ester	0.73±0.11	0.94±0.14	0.61±0.22	1.04±0.34	1.49±0.46
38.59	Stearidonic acid, methyl ester	0.04±0.01	0.07±0.03	0.01±0.01	0.07±0.04	0.08±0.04
38.65	11-(*Z*)-Eicosenoic acid, methyl ester	0.06±0.02	0.08±0.02	0.03±0.01	0.09±0.03	0.12±0.07
38.79	13-(*Z*)-Eicosenoic acid, methyl ester	0.02±0.01	0.03±0.01	0.01±0.01	0.04±0.02	0.05±0.03
39.24	8,11-Eicosadienoic acid, methyl ester	0.01±0.01	0.02±0.01	0.02±0.01	0.02±0.01	0.03±0.02
39.39	11,13-Eicosadienoic acid, methyl ester	0.01±0.01	0.01±0.01	0.05±0.02	0.01±0.01	0.01±0.01
39.51	11,14-Eicosadienoic acid, methyl ester	0.08±0.02	0.11±0.05	0.03±0.02	0.12±0.06	0.15±0.08
39.64	5,8,11-Eicosatrienoic acid, methyl ester	0.10±0.04	0.14±0.06	0.07±0.02	0.16±0.07	0.18±0.10
40.05	Dihomo-gamma-Linolenic acid, methyl ester	0.87±0.11	1.00±0.14	0.50±0.12	1.10±0.33	1.47±0.52
40.47	Arachidonic acid, methyl ester	6.51±1.33	5.98±0.23	4.59±1.06	6.60±0.99	7.74±1.02
40.84	Eicosatrienoic acid, methyl ester	0.03±0.01	0.03±0.01	0.03±0.01	0.04±0.01	0.04±0.01
41.01	Eicosatetraenoic acid, methyl ester	0.06±0.02	0.07±0.03	0.05±0.02	0.08±0.03	0.14±0.09
41.40	Eicosapentaenoic acid, methyl ester	0.63±0.13	0.70±0.12	0.45±0.12	0.78±0.11	0.99±0.21
42.52	Adrenic acid, methyl ester	0.15±0.09	0.19±0.10	0.10±0.11	0.21±0.09	0.31±0.09
43.32	Docosapentaenoic acid, methyl ester	0.68±0.21	0.88±0.29	0.47±0.23	0.97±0.31	1.18±0.21
43.64	Docosahexaenoic acid, methyl ester	4.28±0.45	3.89±0.65	2.77±0.39	4.29±0.0.57	5.41±0.49

Table S4: Fatty acid profile of blood serum in normal or hypercholesterolemic Wistar rats after treatment *Ilex aquifolium* and terpenoid fraction.

RT	Name	Group				
		**CON**	**CHOL**	***I. aquifolium***	**TERP**	**TERPCHOL**
		**Concentration [mg/dL]**				
18.94	Dodecanoic acid, methyl ester	0.06±0.02	0.03±0.02	0.01±0.01	0.02±0.01	0.04±0.01
24.23	Tetradecanoic acid, methyl ester	0.67±0.11	0.36±0.05	0.29±0.07	0.80±0.11	0.54±0.12
24.42	9-(*Z*)-Tetradecanoic acid, methyl ester	0.01±0.01	0.01±0.00	0.01±0.00	0.01±0.01	0.01±0.0.01
26.84	Pentadecanoic acid, methyl ester	0.19±0.07	0.28±0.03	0.21±0.04	0.63±0.08	0.45±0.09
28.16	10-(*Z*)-Pentadecenoic acid, methyl ester	0.03±0.01	0.04±0.01	0.03±0.01	0.04±0.02	0.07±0.01
28.82	Pentadecanoic acid, 14-methyl-, methyl ester	0.01±0.01	0.01±0.01	0.01±0.01	0.01±0.00	0.02±0.01
29.36	Hexadecanoic acid, methyl ester	20.17±2.04	18.67±0.27	7.60±0.14	27.43±0.61	26.37±4.87
30.14	6-(*Z*)-Hexadecenoic acid, methyl ester	0.13±0.04	0.16±0.02	0.11±0.03	0.15±0.02	0.36±0.08
30.35	9-(*E*)-Hexadecenoic acid, methyl ester	0.11±0.04	0.10±0.01	0.07±0.01	0.78±0.06	0.33±0.10
30.64	11-(*Z*)-Hexadecenoic acid, methyl ester	0.02±0.01	0.03±0.02	0.01±0.01	0.04±0.01	0.06±0.02
31.05	Hexadecanoic acid, 14-methyl-, methyl ester	0.01±0.01	0.02±0.01	0.02±0.01	0.03±0.01	0.05±0.01
32.92	10-(*Z*)-Heptadecenoic acid, methyl ester	0.09±0.01	0.05±0.02	0.02±0.01	0.08±0.0.01	0.12±0.01
34.14	Octadecanoic acid, methyl ester	23.59±2.27	26.57±1.60	8.81±0.11	22.49±0.14	26.38±4.53
34.82	9-(*Z*)-Octadecenoic acid, methyl ester	9.41±0.07	12.25±1.23	4.55±0.11	4.72±0.17	17.93±2.38
35.02	9-(*E*)-Octadecenoic acid, methyl ester	0.39±0.12	0.41±0.29	0.36±0.11	1.24±0.05	1.50±0.42
36.22	Linoleic acid, methyl ester	8.14±0.57	9.31±0.28	2.86±0.06	9.30±0.58	13.20±1.75
36.62	10-(*E*)-Nonadecenoic acid, methyl ester	0.15±0.07	0.18±0.22	0.13±0.04	0.01±0.01	0.01±0.01
38.08	9,12-(9*E*,12*E*)-Octadecadienoic acid, methyl ester	0.24±0.08	0.34±0.03	0.21±0.02	0.18±0.04	0.57±0.08
38.50	9,12-(9*Z*,12*Z*)-Octadecadienoic acid, methyl ester	0.08±0.02	0.13±0.01	0.09±0.02	0.07±0.01	0.31±0.12
39.92	8,11,14-Eicosatrienoic acid, methyl ester	0.09±0.04	0.07±0.03	0.03±0.02	0.12±0.01	0.14±0.02
40.31	Arachidonic acid methyl ester	2.22±0.37	2.18±0.27	1.33±0.32	1.62±0.12	2.38±0.27
40.67	Heneicosanoic acid, methyl ester	0.10±0.01	0.10±0.01	0.06±0.01	0.08±0.01	0.19±0.04
41.07	Docosanoic acid, methyl ester	0.05±0.02	0.04±0.02	0.03±0.01	0.06±0.03	0.02±0.02
42.61	4,7,10,13,16,19(all-*Z*)-Docosahexaenoic acid, methyl ester	0.07±0.04	0.08±0.03	0.09±0.03	0.12±0.02	0.12±0.03

Table S5: Fatty acid profile of urine in normal or hypercholesterolemic Wistar rats after treatment *Ilex aquifolium* and terpenoid fraction.

RT	Name	Group				
		**CON**	**CHOL**	***I. aquifolium***	**TERP**	**TERPCHOL**
		**Concentration [mg/dL]**				
18.73	Dodecanoic acid, methyl ester	0.08±0.02	0.18±0.07	0.09±0.02	0.21±0.09	0.34±0.17
24.19	Tetradecanoic acid, methyl ester	0.42±0.12	0.86±0.19	0.43±0.12	0.53±0.15	1.47±0.34
26.80	Pentadecanoic acid, methyl ester	0.04±0.01	0.06±0.02	0.04±0.01	0.08±0.03	0.08±0.02
28.12	10-(*Z*)-Pentadecenoic acid, methyl ester	0.03±0.01	0.05±0.01	0.03±0.01	0.08±0.02	0.08±0.03
28.37	Pentadecanoic acid, 14-methyl-, methyl ester	0.13±0.05	0.27±0.11	0.11±0.03	0.58±0.19	0.68±0.14
29.33	Hexadecanoic acid, methyl ester	7.76±1.99	11.76±2.53	6.74±1.34	10.58±2.01	26.59±3.01
30.31	9-(*E*)-Hexadecenoic acid, methyl ester	0.08±0.04	0.13±0.09	0.05±0.02	0.13±0.08	0.28±0.06
33.20	Heptadecanoic acid, 16-methyl-, methyl ester	0.08±0.03	0.19±0.05	0.07±0.02	0.31±0.12	0.43±0.12
34.11	Octadecanoic acid, methyl ester	30.94±3.21	28.37±4.16	26.07±3.99	26.24±3.12	42.28±2.76
34.79	9-(*Z*)-Octadecenoic acid, methyl ester	0.71±0.13	1.55±0.82	0.66±0.07	1.06±0.09	3.59±1.16
34.98	9-(*E*)-Octadecenoic acid, methyl ester	0.62±0.15	1.87±0.22	0.71±0.11	1.54±0.13	4.16±1.01
36.18	Linoleic acid, methyl ester	0.30±0.11	0.73±0.22	0.24±0.08	0.98±0.22	1.62±0.42
38.57	9,12-(9*Z*,12*Z*)-Octadecadienoic acid, methyl ester	0.03±0.01	0.08±0.04	0.03±0.01	0.14±0.08	0.15±0.05
39.31	Methyl 18-methylicosanoate	0.46±0.15	1.25±0.72	0.52±0.13	2.02±0.34	3.24±0.53
39.85	8,11,14-Eicosatrienoic acid, methyl ester	0.39±0.11	1.08±0.33	0.49±0.15	1.68±0.65	2.80±0.44
40.25	Arachidonic acid methyl ester	0.54±0.16	0.97±0.21	0.39±0.09	1.59±0.49	2.58±0.39
40.60	Heneicosanoic acid, methyl ester	0.14±0.05	0.24±0.07	0.14±0.04	0.41±0.23	0.42±0.12
41.55	5,8,11,14,17-(all-*Z*)-Eicosapentaenoic acid, methyl ester,	0.08±0.02	0.23±0.11	0.09±0.02	0.38±0.11	0.57±0.13
41.95	Tricosanoic acid, methyl ester	0.13±0.04	0.35±0.14	0.16±0.03	0.62±0.16	0.96±0.23
42.30	11-(*Z*)-Eicosenoic acid, methyl ester	0.27±0.12	1.53±0.44	0.32±0.11	2.19±0.43	3.00±0.56
42.36	11-(*E*)-Eicosenoic acid, methyl ester	0.78±0.21	2.88±0.34	0.80±0.06	2.94±0.44	4.05±0.38
42.81	4,7,10,13,16,19-(all-*Z*)-Docosahexaenoic acid, methyl ester	0.32±0.11	0.89±0.19	0.25±0.07	1.08±0.33	2.35±0.44
43.44	Methyl 7,10,13,16,19-docosapentaenoate	0.41±0.17	1.01±0.13	0.40±0.09	1.74±0.31	2.85±0.76
43.89	4,7,10,13,16,19-(all-*Z*)-Docosahexaenoic acid, methyl ester	0.12±0.03	0.55±0.09	0.11±0.02	0.85±0.21	0.96±0.23
44.79	Tetracosanoic acid, methyl ester	0.17±0.06	0.62±0.17	0.25±0.03	0.85±0.18	1.79±0.21
